# Adaptive resilience: agarikon mycelium modulates immune responses and provides oxidative stress buffering in human immune cells

**DOI:** 10.3389/fimmu.2026.1946950

**Published:** 2026-09-09

**Authors:** Elizabeth Doar, Jessica Kishiyama, Zolton J. Bair, Paul Stamets, Chase Beathard

**Affiliations:** Department of Research and Development, Fungi Perfecti, LLC, Olympia, WA, United States

**Keywords:** agarikon, cytokine signaling, immune modulation, *laricifomes officinalis*, mushroom mycelium

## Abstract

**Background:**

Agarikon (*Fomitopsis officinalis, syn. Laricifomes officinalis*) is a fungus with millennia of traditional use across many cultures with modern research supporting the antimicrobial, antiviral, anticancer, antioxidant, and immune-modulating properties of both mycelium and fruit body. Due to the slow growth and old-growth forest habitat of agarikon fruit bodies, its mycelium represents an easily cultivated immunomodulating and stress buffering preparation, underscored by recent clinical trials of a blend of agarikon and *Trametes versicolor* mycelium.

**Methods:**

To provide a stronger mechanistic basis for the observed preclinical and clinical effects supported by agarikon mycelium, this preclinical study investigated the transcriptomic effects of agarikon mycelium in human peripheral blood mononuclear cells (PBMCs) under both basal and LPS-stimulated conditions (*padj* < 0.05), alongside evaluations (*p* < 0.05) of antioxidant, iron chelating, and kinase binding activity. Two agarikon fractions were also assessed (*p* < 0.05) for effects on cell viability and proliferation and induction of select cytokine targets.

**Results:**

Under basal conditions, agarikon mycelium selectively engaged the innate immune response through IL-1 and NF-κB axes, balanced by increases in anti-inflammatory mediators such as IL-1RA and decreases in toll-like receptor transcripts. Under LPS-stimulated conditions, this innate immune response was modified, with measured increases in immune effectors (including *TLR5*) observed in response to induced stress, alongside accompanying transcript decreases in cytokine pathways that can overstimulate the immune system.

**Conclusion:**

Overall, agarikon mycelium demonstrated a coordinated, context-dependent immune response profile, supporting its stress-buffering and immune-modulating potential and warranting continued clinical validation.

## 1 Introduction

Agarikon (*Fomitopsis officinalis*, syn. *Laricifomes officinalis*) is a medicinal mushroom with millennia of traditional use in many cultures, with preparations included in ancient pharmacopoeias such as Dioscorides’ De Materia Medica (1–3). Modern research has supported the antimicrobial, antiviral, anticancer, immunomodulatory, and antioxidant properties of individual components and whole extracts of agarikon mycelium (2–12) and fruit body (4–15). The antioxidant and anticancer effects of agarikon treatment are partially enacted by its distinct immunomodulatory capacities and mediated by the nuclear factor kappa-light-chain-enhancer of activated B cells (NF-κB) pathway among others, as demonstrated *in vitro* using cancer cell lines and *in vivo* using zebrafish models (13–15).

Fungi can produce compounds unique to developmental stage, substrate, and extract type, which influence the immunological effects of fungal treatments (16, 17). For instance, two chlorinated coumarins have been isolated from agarikon mycelium, with structure-dependent antibacterial activity against tuberculosis, expanding the reported bioactive metabolite space of this species beyond its well-studied terpenoid fraction (6). Additionally, investigations of the antiviral activity of agarikon have thus far centered on the action of mycelial fractions, in particular against pox viruses (12) and influenza A (18). A specific agarikon fruit body fraction was found to act as a toll-like receptor (TLR) agonist by activating NF-κB and the innate immune system via TLR2 and TLR4, and bolstered the adaptive immune system by binding to and preventing the interaction of programmed cell death protein 1 (PD-1) and its ligand 1 (PD-L1) and vascular endothelial growth factor (VEGF) and its receptor (VEGFR-2), which inhibits tumor angiogenesis (14, 15). A broader fraction of agarikon fruit body was also observed to engage this pathway by upregulating NF-κB inhibitor alpha (*NFKBIA*), NF-κB subunit 1 (*NFKB1*), and transcription factor p65 (*RELA*) protein products in HepG2 cancer cells as part of its anticancer effect (13). However, some preclinical studies suggest that extracts with high concentrations of polysaccharides from fruit bodies of certain species, including *Ganoderma lucidum* and *Hericium erinaceus*, may reduce viability (19) or induce stress responses (17) in human immune cells. Collectively, these findings suggest that immunological activity elicited by fungal-derived preparations is highly dependent on biological source and extraction method, necessitating functional evaluation across tissue types and solvent-based extracts to characterize context-dependent immune responses *in vitro*.

Because agarikon fruit bodies are slow growing and largely limited to old-growth forest habitats, contributing to its Endangered status on the IUCN Red List of Threatened Species, aseptically cultured mycelium has emerged as a controlled and scalable method for accessing its bioactive potential without reliance on wild harvest (11, 20). Accordingly, one cryopreservation program has established a repository containing at least 87 genetically distinct agarikon strains, supporting long-term conservation of its genetic diversity (21). Recently, an annotated nuclear genome of agarikon has been made publicly available for the first time, providing foundational resources for understanding its genetic architecture and biosynthetic potential, and reflecting its status as a phylogenetically distinct polypore and sole species in the genus *Laricifomes* (22). Building on the genetic, ecological, and (pre)clinical functional rationale for investigating agarikon mycelium, the aim of this study was to characterize its potential as a cytocompatible, context-dependent immunomodulatory treatment in PBMCs, using transcriptomic profiling supported by complementary functional assays evaluating redox biology, cytokine responses, kinase binding activity, and antiviral activity.

## 2 Materials and methods

### 2.1 Sample preparation

After confirmation of species identity via DNA sequencing of the internal transcribed spacer (ITS) region (EU854439.1), *TEF1*, and *RPB2*, two different extracts (hydroethanolic: EtOH and ethyl acetate: EtOAc) of agarikon mycelial samples were prepared. Ethanol and ethyl acetate were selected as solvents because they recover complementary classes of fungal metabolites with differing polarity, enabling comparison of chemically distinct fractions across downstream functional assays. EtOH extracts were made by combining 20 g of Host Defense agarikon powder (*F. officinalis* mushroom mycelium and fermented brown rice biomass; Fungi Perfecti, LLC, Olympia, WA, USA) with 50 g 95% ethanol (ThermoFisher, Waltham, MA, USA, T038181000) and 50 g ultrapure water from a Direct-Q™ ultrapure water purification system (Millipore Sigma, Burlington, MA, USA, ZRQSVP3US). The sample was macerated for 120 h at 22°C, centrifuged at 600 x *g* for 10 min using an Eppendorf high speed centrifuge (Enfield, CT, USA, 5810), and the supernatant decanted. Centrifugation was repeated until the pellet was nearly dry. After maximum supernatant recovery, samples were rotary evaporated using a Buchi R-100 (New Castle, DE, USA) to reduce fluid volume and frozen at –80°C (ThermoFisher, TSX50086CPM). Samples were then lyophilized to dryness in a Labconco FreeZone (Kansas City, MO, USA, 720402000), transferred to a new sterile tube, and stored at 22°C. Then, samples were reconstituted in water, DMSO (VWR, Radnor, PA, USA), or PBS (Gibco, Grand Island, NY, USA, 10010031), passed through a 0.2 μm polypropylene filter (PALL Life Sciences, Port Washington, NY, USA, 4433), and used for functional assays. EtOAc samples were prepared by milling lyophilized agarikon mycelium to reduce particle size. 2 g of milled agarikon powder was combined with 10 mL of ethyl acetate (Tedia, Fairfield, OH, USA, DES15120-4Y), sonicated for 10 min, vortexed for 30 min, and set to macerate for 24 h at 22°C. After maceration, the sample was centrifuged at 600 x *g* for 10 min, and the supernatant decanted. Centrifugation was repeated until the pellet was nearly dry. After maximum supernatant recovery, the samples were rotary evaporated to remove any excess solvents and lyophilized to dryness. Samples were reconstituted in DMSO and passed through a 0.2 μm polypropylene filter before being used for functional assays.

### 2.2 Cell viability and proliferation testing

#### 2.2.1 MTT evaluation of cell viability and cytotoxicity

Before culturing PBMCs for RNA extraction and additional assays, MTT assays (ATCC, Manassas, VA, USA, 30-1010K) were performed to evaluate cell viability (as based on metabolic activity) following treatment with agarikon extract preparations. MTT assays were performed in accordance with the manufacturer’s kit instructions, but with a decrease in volume from 110 μl to 55 μl reactions to conduct the assay in a 384-well plate (ThermoFisher, 464718), with a minimum of n = 4 replicate wells per treatment concentration. Standard human PBMCs (ATCC, PCS-800-011) were thawed, centrifuged, and washed with 37°C RPMI 1640 media (ATCC, 30-2001) to remove DMSO used for cryopreservation purposes. Cells were incubated in a Heracell VIOS CO_2_ incubator (ThermoFisher, 51032874) in 15 mL of warmed RPMI 1640 (10% FBS, heat inactivated; Sigma-Aldrich, St. Louis, MO, USA, F1051-100ML) overnight in a T75 flask (Nest Scientific, Woodbridge, NJ, USA) at 37°C in a 5% CO_2_ atmosphere to recover from the freeze/thaw process. Cell counts were determined via hemocytometer (LW Scientific, Lawrenceville, GA, USA, CTL-HEMM-GLDR) after staining with 0.4% trypan blue (HiMedia, Kennett Square, PA, USA, GRM263) diluted in PBS. Following a 24 h growth phase within the flask, cells were transferred to a 96-well plate (2 × 10^6^ cells per well; Nest Scientific, 701001). Treatments were introduced approximately 24 h after seeding, and plates were incubated for 18–24 h post-treatment before addition of the MTT reagent and further incubating at 37°C in a 5% CO_2_ atmosphere for 4–24 h to facilitate formazan crystal formation. Detergent was then added for 4–24 h until crystals were completely solubilized. Absorbance readings at 570 and 660 nm and all other plate-based assay data were recorded using a BioTek Synergy HTX microplate reader (Agilent Technologies, Santa Clara, CA, USA). Sample values were calculated by subtracting values at 660 nm from values at 570 nm. Percent cell viability was evaluated relative to either the 0.2% PBS or 0.2% DMSO vehicle controls, according to each sample’s vehicle solvent.

#### 2.2.2 Fluorometric evaluation of cell proliferation

Cells were also evaluated for cellular proliferation levels under both basal and LPS inflammatory conditions via fluorometric assessment using CyQUANT cell proliferation assay components (Invitrogen, Waltham, MA, USA, C35006) to more thoroughly explore the cytocompatibility of agarikon mycelium carried in a variety of solvents in human cell culture. Standard human PBMCs were cultured and treated as described for the MTT assay and then centrifuged at 100 x *g* for 6 min. Cell media was aspirated and frozen at –20°C for use in ELISA quantification. A working solution of 1X HBSS buffer containing CyQUANT NF dye reagent (1:500 dilution) was prepared in accordance with the nonadherent cell protocol provided. Cells were washed with PBS and aspirated before 100 µL of combined 1X HBSS buffer and dye reagent was added to each well. Plates (Invitrogen, M33089) were incubated at 37°C for 60 min before being read fluorometrically (485 nm excitation, 528 emission), and cell proliferation values were calculated as percentages relative to the 0.2% DMSO vehicle control.

#### 2.2.3 Cell imaging

To produce representative images of cells for MTT and fluorometric cell proliferation assays, pictures of agarikon- and vehicle-treated cells were obtained either by staining with 0.4% trypan blue diluted in PBS and imaging directly using a Leica DMi1 microscope (Deerfield, IL, USA) and Leica MC170 HD digital microscope camera, or by resuspending cells in 1X HBSS buffer and dye binding solution (Invitrogen, C35006) and imaging with the Leica DMi1 and Leica MC170 under UV illumination, adapted from (23). ImageJ (v1.54p) was used for analysis.

### 2.3 PBMC culture for mRNA-sequencing

mRNA-sequencing (RNA-Seq) was used to assess global gene expression changes in PBMCs following agarikon treatment under basal and LPS-stimulated conditions. After an initial 24 h incubation in 12-well plates (ThermoFisher, FB012928), PBMCs were treated and incubated for a further 24 h, including 100 ng/mL LPS (Millipore Sigma, Burlington, MA, USA, L4391-1MG) for inflammatory condition samples. For harvest, cells and media were aspirated, pipetted into sterile 1.5 mL microcentrifuge tubes (Eppendorf, 05-402-25), washed with PBS, and aspirated again before freezing cell pellets in liquid nitrogen prior to storage at –80°C.

Before starting RNA extraction, surfaces and tools were cleaned with RNaseZap (ThermoFisher, AM9780). Using the RNeasy Mini Kit (Qiagen, Germantown, MD, USA, 74104), 350 µL of RLT buffer was added to each microcentrifuge tube after flicking to loosen the cell pellet. Cells were lysed by vortexing for 1 min, and the supernatant was transferred to a new sterile 1.5 mL microcentrifuge tube. The manufacturer’s recommended extraction procedure for mammalian cells was followed, including the optional step of placing columns into new collection tubes and centrifuging (ThermoFisher, 13-100-675) for 1 min at 13,000 x *g* immediately before elution. For elution, 30 µL of RNase-free water was used, and columns were incubated at 22°C for 1 min before proceeding. RNA quality (A260/280 ratios) and concentrations of samples were determined spectrophotometrically prior to DNase digestion, where 30 µL of eluted RNA was added to 3 µL of reaction buffer and 3 µL of DNase I (ThermoFisher, EN0521). Samples were incubated at 37°C for 30 min before 3 µL of 50 mM EDTA (ThermoFisher, EN0521) was added, then incubated at 65°C for 10 min. All samples were stored at –80°C and shipped to Novogene Corporation Inc. (Sacramento, CA, USA) on dry ice for RNA-Seq.

### 2.4 RNA-seq and analysis

Extracted samples proceeded to sequencing if they had a total RNA quantity of ≥100 ng and a spectrophotometric A260/280 ratio of 1.95 or greater. Novogene’s mRNA-Seq service was used with poly(A) enrichment and performed on an Illumina NovaSeq X-Plus platform with a paired-end read length of 150 bp. At least 20 million read pairs per sample were generated and aligned to the reference genome *Homo sapiens* GRCh38 (hg38); RNA-Seq data are summarized in **Supplementary Tables 1**, **S2**. NovoMagic (Novogene’s RNA-Seq platform) was used to generate differentially expressed gene (DEG) lists and dot plots summarizing KEGG and Reactome pathways. DESeq2 analysis, applying the Benjamini-Hochberg false discovery rate (FDR) correction with an adjusted *p*-value (*p*adj) cutoff of ≤ 0.05, was used to identify significant DEG and pathway results (24). PCAGO was used to assess variability via principal component analysis (PCA), RStudio 2026.05.0 (R 4.6.0) was used to create bar and line graphs, volcano plots, and heatmaps, and KEGG Mapper – Color was used to generate and color code KEGG plots.

### 2.5 Cytokine activity assays

Protein-level confirmation of select RNA-Seq outcomes was pursued using ELISAs to provide a complementary evaluation of the functional immune response in PBMCs. Standard human PBMCs were cultured as described for evaluation of cell proliferation and viability, and conditioned cell media was aspirated and used for cytokine analysis via ELISA. Cytokine concentrations were quantified using commercially available ELISA kits for interleukin 8 (IL-8; R&D Systems, Minneapolis, MN, USA, DY208-05), interleukin-1 receptor antagonist (IL-1RA; R&D Systems, DY280-05), and interleukin-1 alpha (IL-1α; R&D Systems, DY200-05). All ELISAs were run according to manufacturers’ instructions besides volume modifications to accommodate a 384-well format. IL-8 and IL-1RA used 0.2% PBS and 0.2% DMSO as vehicle controls, and IL-1α used LPS as a vehicle control.

### 2.6 MAPK binding assays

Investigation of the mitogen-activated protein kinase (MAPK) binding activity of agarikon mycelium was performed using Eurofins’ (San Diego, CA, USA) DiscoverX KINOMEscan profiling service to explore secondary signaling pathways induced by this strain of agarikon that could influence immune responses. An EtOAc extract was selected to enrich for hydrophilic and lipophilic secondary metabolites. c-Jun N-terminal kinase 3 (JNK3) and tropomyosin receptor kinase A (TRKA) were included in KINOMEscan, a competitive binding assay used to evaluate the ability of agarikon mycelium extracts to disrupt interactions between kinases and immobilized ligands. Results are reported as percent control values, where lower values indicate stronger kinase binding interactions relative to the DMSO control (25, 26).

### 2.7 DPPH assays

DPPH radical scavenging assays were employed to assess the antioxidant potential of agarikon and provide functional corroboration of the reactive oxygen stress- and redox-related pathways explored via transcriptomics. A 100 µg/mL DPPH reagent (Sigma-Aldrich, 300267) stock was prepared in 99% ethanol and calibrated by adding 60 µL of DPPH stock to 40 µL of sterile water in a single well of a 384-well plate and ensuring absorbance values fell between 1.0 and 1.1 when read spectrophotometrically at 517 nm. DPPH reagent was included as a maximal signal control, and agarikon treatment was serially diluted in sterile water or 99% ethanol before being added to a 384-well plate at a volume of 75 µL per well. Background was evaluated at 517 nm before adding 25 µL DPPH stock to each well and reading at 517 nm every 5 min for 60 min, shaking between read points. Final absorbance values were obtained by subtracting the average background absorbance at 60 min from all other wells, and percent radical scavenging activity was calculated by subtracting sample absorbance from the maximal signal control, dividing by the maximal signal control, and multiplying by 100, where the maximal signal control was defined as the absorbance of DPPH reagent alone at 60 min.

### 2.8 Evaluation of ferrous iron chelating ability

In addition to antioxidant assessment and transcriptomic analysis of iron homeostasis-related signaling pathways, the iron chelation activity of agarikon mycelial extract was measured to directly quantify its iron-sequestering potential. Following (27) and (28), ferrous iron (Fe²^+^) chelating activity was measured by quantifying the reduction in color intensity after inhibition of the purple ferrozine–Fe²^+^ complex. Water was used for all sample and component dilutions and controls, and an EDTA serial dilution (100 µM-0.78 µM) was included to express iron chelation ability in EDTA µM equivalents. In 1.5 mL microcentrifuge tubes, 500 µL of control, blank, or sample was added to 900 µL of sterile water, and 100 µL of 0.6 mM FeCl_2_ (ThermoFisher, 389350250) was added to all reactions except the blank, where it was replaced with 100 µL water. Reaction tubes were vortexed, incubated for 10 min at 22°C with additional vortexing every 2 min, and then 100 µL 5 mM ferrozine (ThermoFisher, AC410570010) was added to all reactions in the dark. Vortexing was repeated before transferring reactions to a 96-well plate (200 µL per replicate well; ThermoFisher, 442404), which was incubated for 10 min at 22°C in the dark before measuring absorbance spectrophotometrically at 562 nm. To determine the ferrous iron chelating percentage of samples, the average blank absorbance was used for correction before subtracting sample absorbance from the average maximal signal control value, dividing by average maximal signal control value, and multiplying by 100. An EDTA standard curve was constructed and used to determine EDTA µM equivalents for each sample.

### 2.9 Antiviral activity assays

Because of consistent viral-related RNA-Seq pathway responses and past reports of agarikon as an effective antiviral treatment in both *in vitro* and *in vivo* contexts (9, 10, 12, 18), additional support for the antiviral activity of this agarikon strain became increasingly relevant. Extracts of agarikon mycelium have previously been evaluated for activity against an array of viruses as part of Project BioShield (12), including the strain used in the current study, which was tested as part of this program and follows methods reported in (12). In brief, EtOH extracts of agarikon mycelium were tested for antiviral activity in human cells against a wide range of viruses, including influenza A, using a neutral red assay and a visual cytopathic effect assay. Results are reported compared to the control antiviral drug ribavirin.

## 3 Results

### 3.1 Agarikon mycelium promotes cellular metabolic activity and proliferation across basal and LPS-stimulated conditions

**Figure 1:**
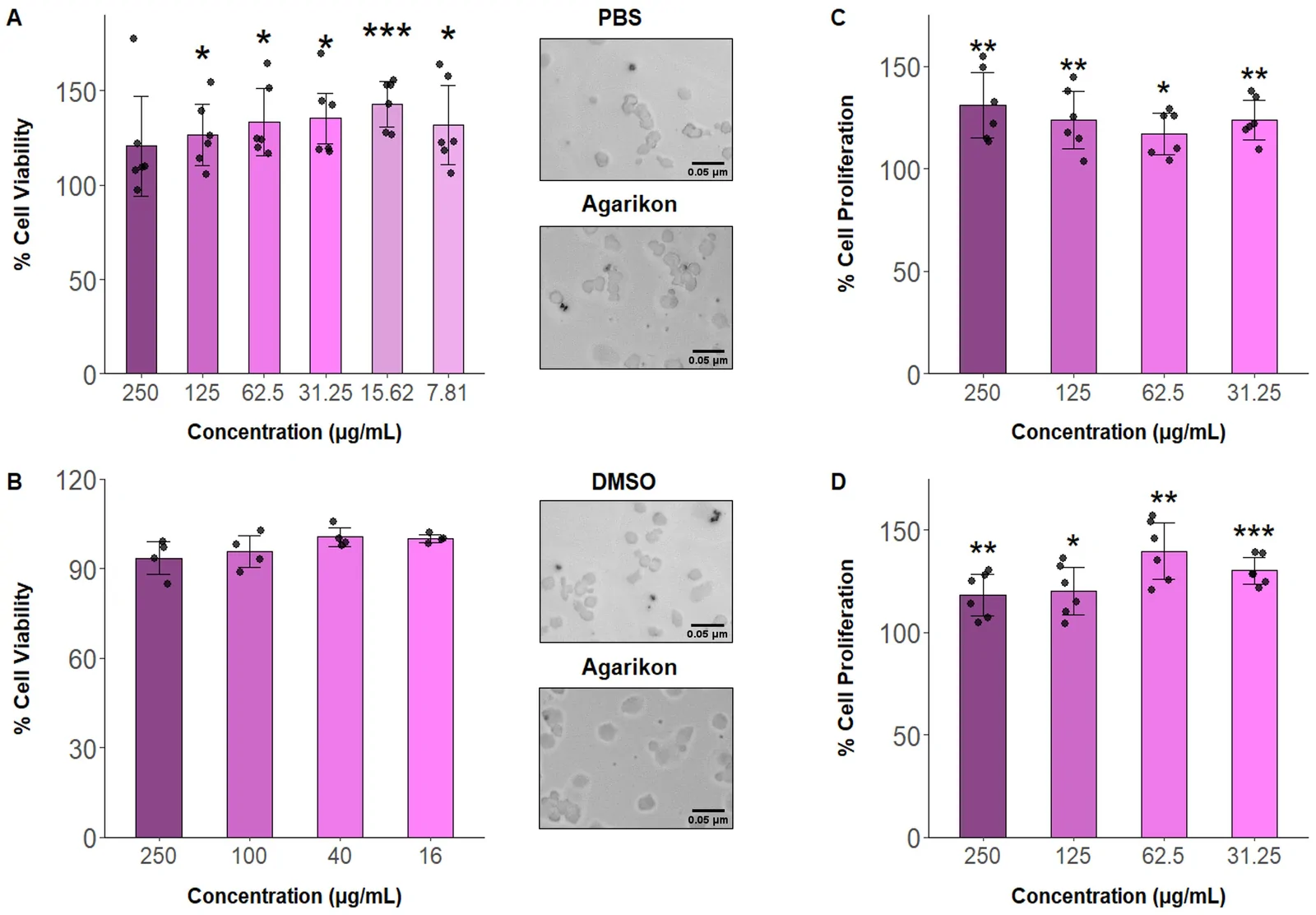
Percent cell viability of human peripheral blood mononuclear cells (PBMCs) treated with **(A)** hydroethanolic (EtOH) or **(B)** ethyl acetate (EtOAc) extracts of agarikon (*Fomitopsis officinalis*, syn*. Laricifomes officinalis*) mycelium, as evaluated by MTT assay. **(C)** Fluorometric assay of cell proliferation after treatment with an EtOH agarikon fraction relative to the vehicle (DMSO) under basal conditions and **(D)** LPS-stimulated conditions relative to vehicle with LPS. In all cases, n ≥ 4 wells per treatment concentration; significance evaluated relative to vehicle controls, two-tailed t-test, unequal variances, *p < 0.05; **p < 0.01; ***p < 0.001.

To assess cytocompatibility and support selection of treatment concentrations, cell metabolic activity and proliferation were evaluated using MTT and fluorometric assays, respectively (**Figure 1**). The EtOH fraction of agarikon mycelium resulted in a consistent increase in cell viability, with the five lowest concentrations resulting in significant metabolic increases of at least 20% relative to the vehicle (**Figure 1A**), while an EtOAc fraction was observed to maintain viability, but not promote augmented metabolic outcomes (**Figure 1B**). Following treatment with the EtOH fraction cellular proliferation was maintained under both basal and LPS-stimulated conditions, with statistically significant increases compared to the vehicle observed across all tested concentrations (**Figures 1C, D**). These findings were supported by morphological observations of agarikon-treated cells (**Supplementary Figure 1**).

### 3.2 Context-dependent transcriptomic signatures of immune and stress responses

**Figure 2:**
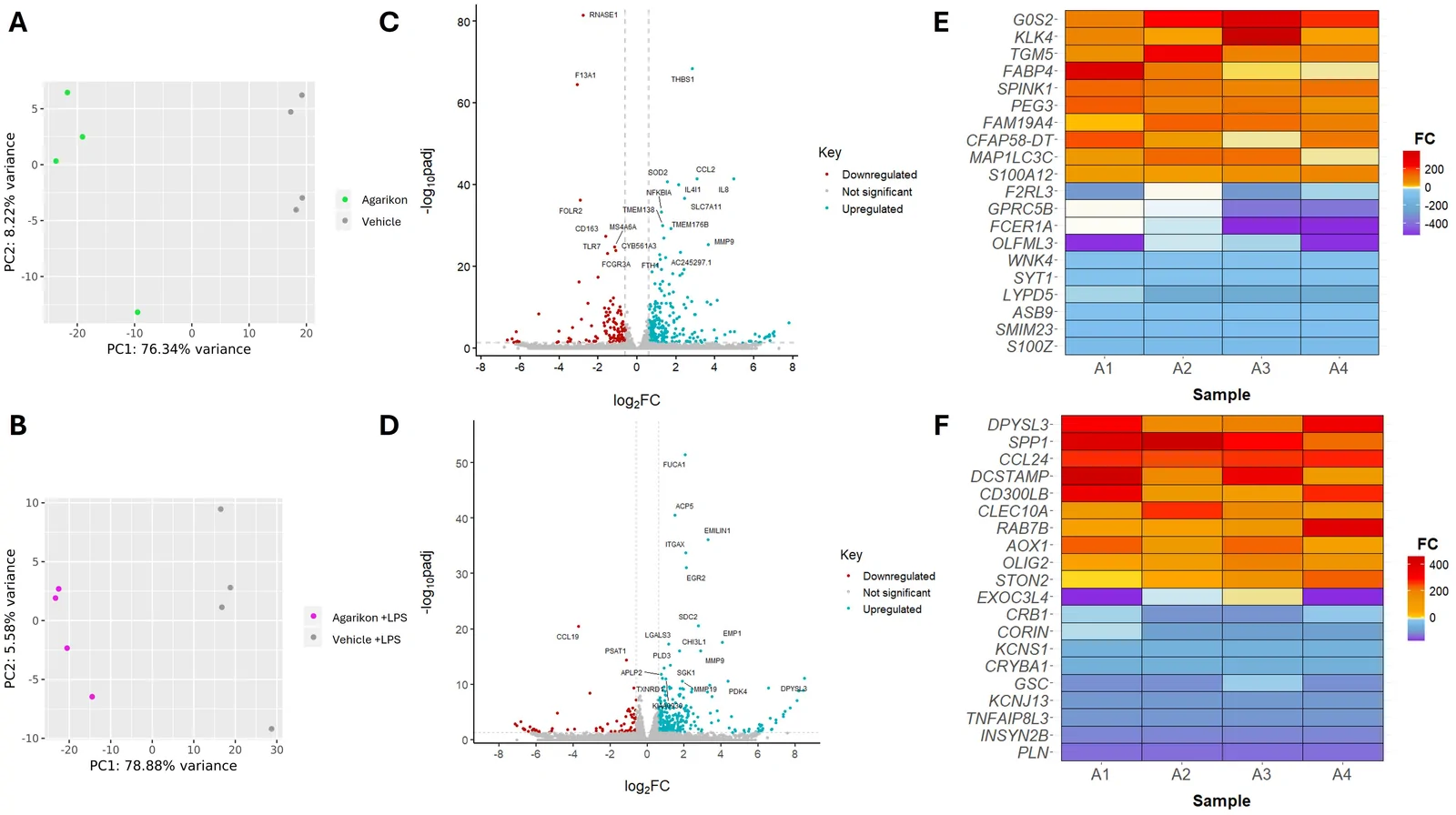
**(A)** Principal component analysis (PCA) for agarikon- and vehicle-treated samples under basal and **(B)** inflammatory conditions evaluating the similarities or differences between these treatment groups based on their transcriptomic clustering patterns (n = 4). **(C)** Volcano plot for agarikon-treated PBMCs relative to Vehicle under basal and **(D)** inflammatory conditions, displaying the magnitude of differential gene expression (log_2_FoldChange) in relation to statistical significance [-log_10_(*p*adj)]. Statistically significant results are indicated in blue for downregulated genes and red for upregulated genes (n = 4, DESeq2, *p*adj < 0.05). **(E)** Top 10 up- and down-regulated genes by fold change (FC) for the agarikon treatment relative to Vehicle under basal and **(F)** inflammatory conditions (n = 4, DESeq2, *p*adj < 0.05).

PCA was performed for both basal and inflammatory RNA-Seq data to compare gene expression trends, revealing that agarikon treatment samples clustered separately from vehicle samples under both basal and inflammatory conditions (**Figures 2A, B**). PC1 under both basal (76.34%) and inflammatory (78.88%) conditions accounted for the majority of variance, while PC2 contributed minimally (8.22% and 5.58%, respectively). In both cases, the transcriptome was largely organized along a single dominant axis of variation, consistent with a strong treatment-associated transcriptional outcome.

Consistent with the clear treatment-associated separation observed by PCA, wide-ranging transcriptional differences were revealed between agarikon treatment relative to Vehicle (DESeq2 analysis, *p*adj < 0.05, **Figure 2C**). Under basal conditions, 351 genes were upregulated and 184 genes were downregulated. The most statistically significant results included ribonuclease A family member 1, pancreatic (*RNASE1*, *p*adj < 3.98 × 10^-82^, FC = -6.87), *CXCL8*/*IL8* (*p*adj < 3.89 × 10^-42^, FC = 31.02), superoxide dismutase 2 (*SOD2*, *p*adj < 1.75 × 10^-41^, FC = 2.93), interleukin 4 induced 1 (*IL4I1*, *p*adj < 1.07 × 10^-40^, FC = 4.37), and *NFKBIA* (*p*adj < 4.70 × 10^-34^, FC = 2.39). Highly upregulated transcripts included G0/G1 switch 2 (*G0S*, FC = 220.55), kallikrein related peptidase 4 (*KLK4*, FC = 130.18), and transglutaminase 5 (*FGM5*, FC = 126.47), while the most strongly downregulated transcripts included S100 calcium binding protein Z (*S100Z*, FC = -101.90), LY6/PLAUR domain containing 5 (*LYPD5*, FC = -74.56), and Fc fragment of IgE receptor Ia (*FCER1A*, FC = -16.50; **Figure 2E**).

Under inflammatory conditions, agarikon treatment upregulated 437 genes, representing a greater transcriptional activation response than observed under basal conditions, while a comparable number of genes were downregulated (181 genes). Among the most statistically significant results (**Figure 2D**) were alpha-L-fucosidase 1 (*FUCA1*, *p*adj < 2.99 × 10^-52^, FC = 4.12), C-C motif chemokine ligand 19 (*CCL19, p*adj < 3.76 × 10^-21^, FC = -13.07), galectin 3 (*LGALS3*, *p*adj < 5.04 × 10^-18^, FC = 2.24), and thioredoxin reductase 1 (*TXNRD1*, *p*adj < 7.77 × 10^-12^, FC = 1.75). Highly upregulated results included dihydropyrimidinase like 3 (*DPYSL3*, FC = 357.89), C-C motif chemokine ligand 24 (*CCL24*, FC = 292.25), and C-type lectin domain containing 10A (*CLEC10A*, FC = 173.84), whereas the most downregulated results included phospholamban (*PLN*, FC = -131.90), inhibitory synaptic factor family member 2B (*INSYN2B*, FC = -114.56), and TNF alpha induced protein 8 like 3 (*TNFAIP8L3*, FC = -100.04; **Figure 2F**).

**Figure 3:**
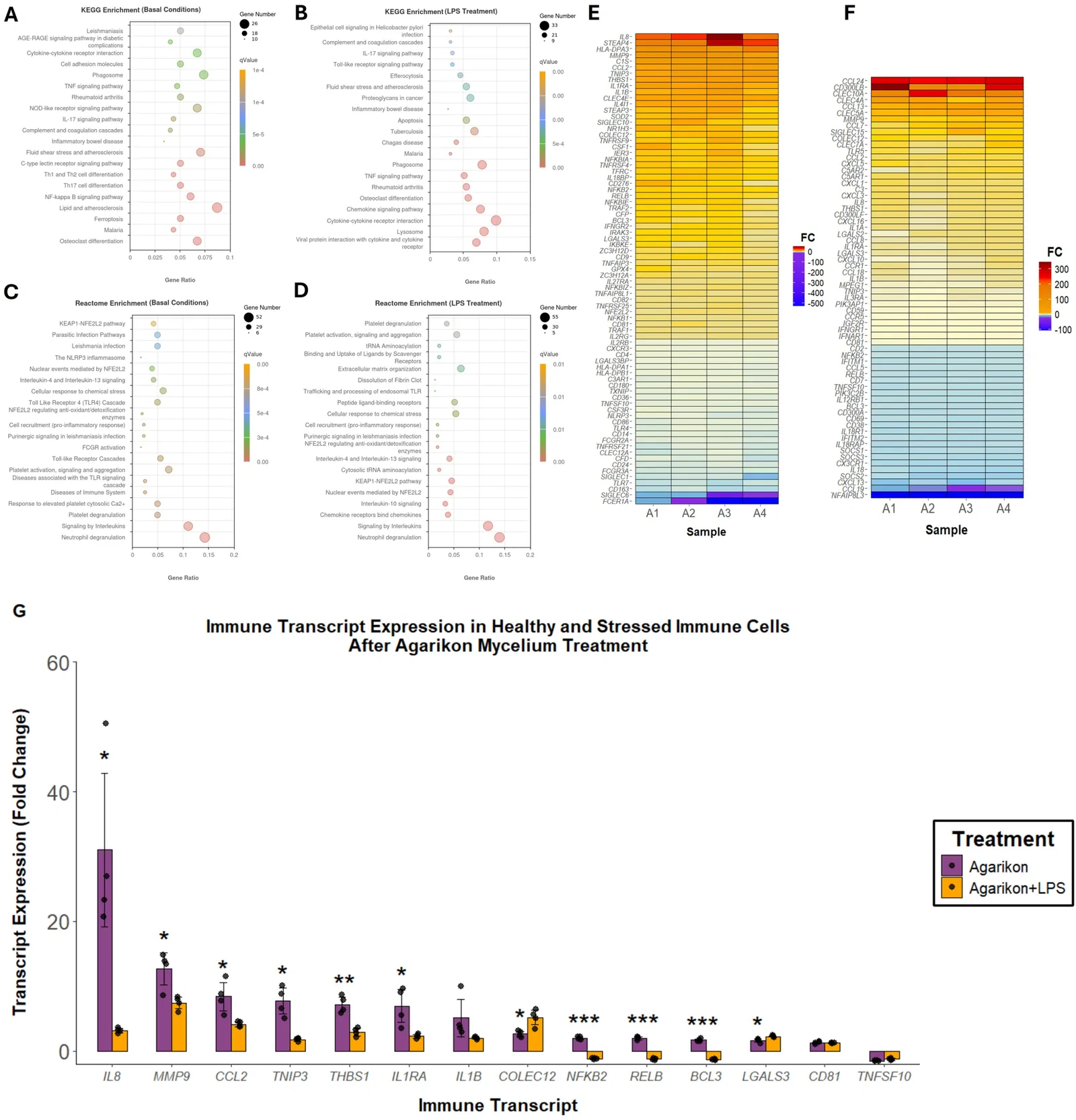
**(A)** KEGG dot plot of enriched pathways in agarikon-treated PBMCs relative to Vehicle under basal and **(B)** inflammatory conditions (n = 4, DESeq2, *p*adj < 0.05). **(C)** Reactome dot plot of enriched pathways in agarikon-treated PBMCs relative to Vehicle under basal and **(D)** inflammatory conditions (n = 4, DESeq2, *p*adj < 0.05). **(E)** Select immune-related differentially expressed genes (DEGs) identified in PBMCs treated with agarikon compared to Vehicle under basal and **(F)** inflammatory conditions (n = 4, DESeq2, *p*adj < 0.05). **(G)** Comparative levels of select immune DEGs shared by basal and inflammatory conditions (n = 4, significance testing performed by comparing basal condition averages to inflammatory condition averages, two-tailed t-test, unequal variances, *p < 0.05; **p < 0.01; ***p < 0.001). Complete lists of immune DEGs under basal and LPS-inflammatory conditions can be found as **Supplementary Tables 3**, **S4**.

Under basal conditions, significantly (*p*adj < 0.05) affected pathways in agarikon-treated PBMCs clustered around degranulation processes, TLR and interleukin immune signaling, and antioxidant/detoxification pathways (**Figures 3A, C**). After LPS stimulation, these pathways centered on appropriate response to simulated microbial invasion, including cytokine and TLR signaling and degranulation processes, while retaining signs of antioxidant and anti-inflammatory pathway engagement (**Figures 3B, D**). Individual transcript responses were consistent with these pathway results. Notable immune DEGs upregulated under basal conditions included *CXCL8/IL8, IL1RN/IL1RA*, IL-1 beta (*IL1B*)*, IL4I1, SOD2*, IL-18 binding protein (*IL18BP*), NF-κB subunit 2 (*NFKB2*), transcription factor RelB (*RELB*) and several cluster of differentiation (CD) markers, though *TLR4* and *TLR7* are significantly downregulated. While *IL8* transcript fold change (FC) values are robust, actual protein induction is much lower, with no agarikon treatment increasing IL-8 protein by more than 30 pg/mL (**Supplementary Figure 2**). Under inflammatory conditions*, IL8, IL1RA*, and *IL1B* were also upregulated, though not to the degree observed under basal conditions. In this challenged environment, while *TLR5, IL1A*, and many CD transcripts increased, they were attenuated in comparison to basal conditions, and opposite to basal conditions, *IL18* and the noncanonical NF-κB subunits (*NFKB2* and *RELB*) were downregulated (**Figures 3E, F**).

When comparing FC values of immune DEGs in response to agarikon treatment under basal and inflammatory conditions, significant trends emerge (**Figure 3G**). Human PBMCs in LPS-induced inflammatory conditions produced ten-fold less *IL8* after treatment with agarikon compared to basal PBMCs, alongside a non-significant decrease in *IL1B* (*p* < 0.05). This is paired with a significant decrease in levels of *IL1RA*, as well as the active downregulation of *NFKB2* and *RELB*, key subunits of the noncanonical NF-κB pathway. Additionally, under basal conditions, the upregulation of immunoprotective factors such as *IL18BP* and *SOD2* is paired with the downregulation of key inflammatory actors such as *TLR4, TLR7*, and *CD14*. Agarikon treatment was associated with condition-dependent differential expression of TLR genes, including decreased *TLR4* and *TLR7* under basal conditions and increased *TLR5* under LPS-stimulated conditions, consistent with KEGG pathway annotations of upstream pattern recognition signaling components (**Supplementary Figure 3**). Overall, agarikon appears to induce context-dependent modulation of immune-associated gene expression under both basal and LPS-stimulated conditions.

### 3.3 Antioxidant capacity of agarikon mycelium

**Figure 4:**
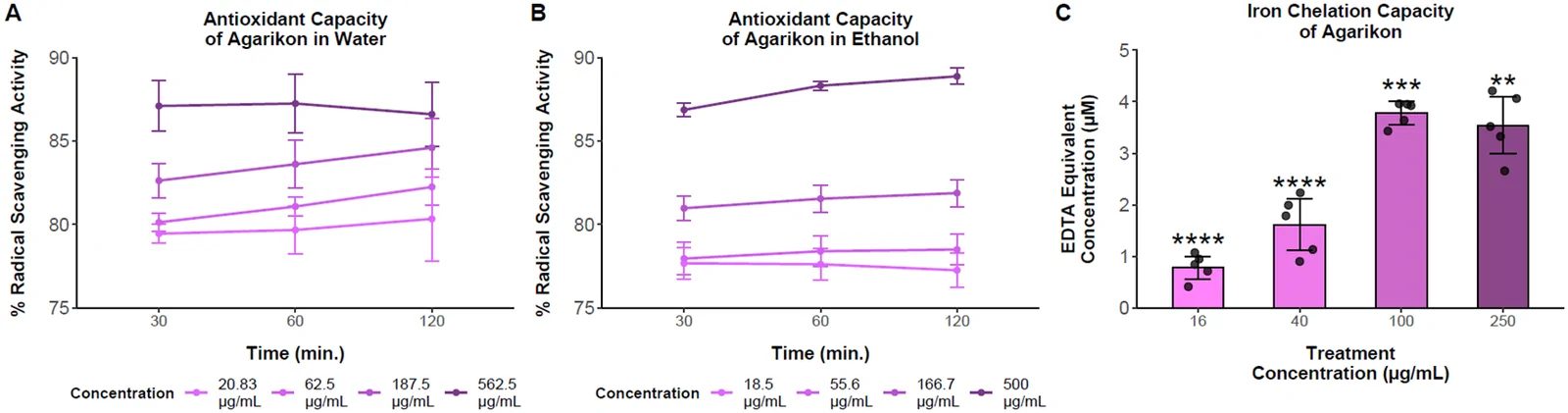
**(A)** Antioxidant capacity of agarikon over time in water and **(B)** ethanol as evaluated by DPPH assay (n = 7). **(C)** Ferrous iron chelation capacity of agarikon, expressed as EDTA concentration equivalents (n = 5; significance testing performed by comparing to maximum signal control, two-tailed t-test, unequal variances, **p < 0.01; ***p < 0.001; ****p < 0.0001).

Based on the transcript- and pathway-level antioxidant and ferroptosis results observed after agarikon treatment, DPPH radical scavenging activity (RSA) and ferrous iron chelating ability were also investigated (**Figure 4**). Agarikon mycelial extract exhibited significant, dose-dependent RSA in both water and ethanol extracts, and the response occurred rapidly, with >75% of total RSA achieved within 30 minutes. Activity was maintained across a broad concentration range, from 500 µg/mL to a minimum of 20.83 µg/mL (resuspended in water) and 18.5 µg/mL (resuspended in ethanol). Average RSA for this treatment was slightly higher when resuspended in ethanol, approaching 90% of the maximal control. Ferrous iron chelation activity also increased in a dose-dependent manner, with all tested concentrations displaying significantly more iron chelation activity than the maximal signal control (*p* < 0.05; **Figure 4C**). The magnitude of this response increased approximately four-fold across the concentration range tested before approaching a plateau at the two highest concentrations.

### 3.4 Cytokine modulation and secondary immune signaling

**Figure 5:**
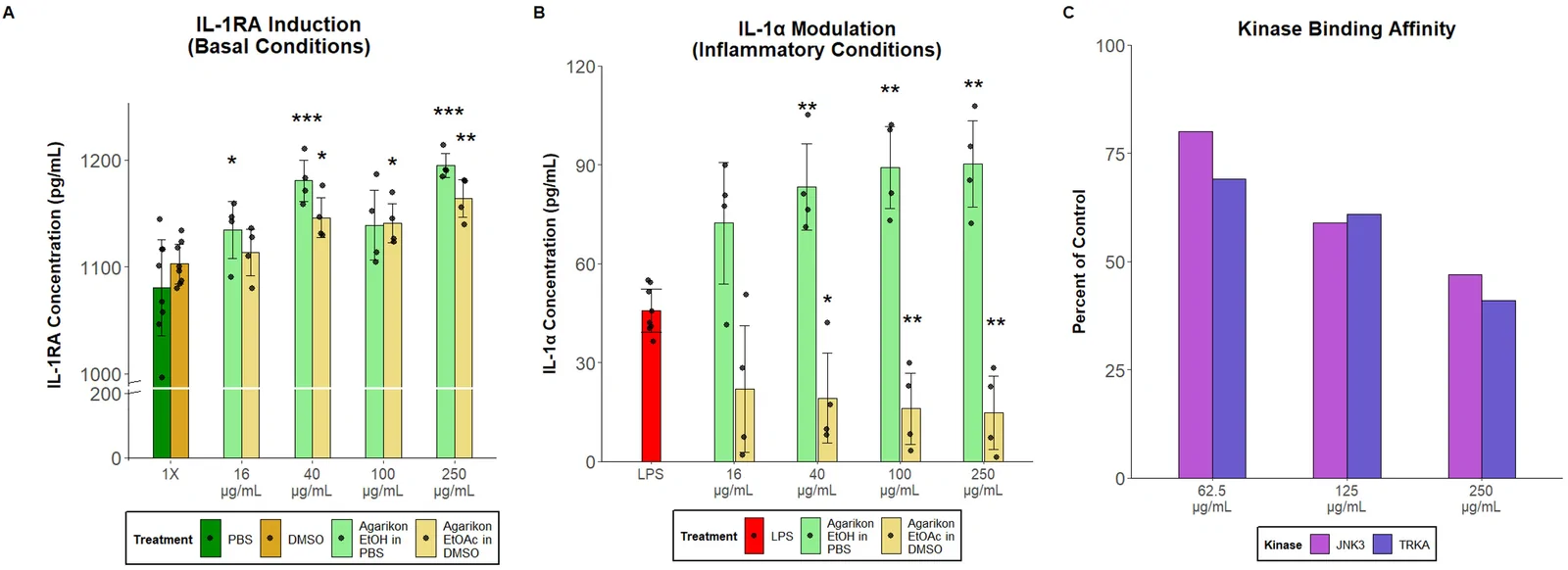
**(A)** IL-1RA cytokine responses in agarikon-treated human PBMCs under basal conditions (n = 4). **(B)** IL-1α responses under inflammatory conditions (n = 4). In all cases, significance was evaluated relative to respective vehicle controls (PBS, DMSO, or LPS) using two-tailed t-tests with unequal variances (*p < 0.05; **p < 0.01; ***p < 0.001). **(C)** Kinase binding affinity of agarikon mycelium treatment toward JNK3 and TRKA as evaluated by Eurofins’ DiscoverX KINOMEscan profiling service. Lower values indicate greater engagement of the kinase by treatment and higher binding affinity overall.

To provide protein-level assessment of transcriptomic immune responses, ELISAs for select cytokine targets were also performed. Similar to transcript-level findings, the EtOH agarikon preparation induced significant increases in IL-1RA under basal conditions, while a similar induction was also observed for the EtOAc fraction (**Figure 5A**). Under inflammatory conditions, both the EtOH and EtOAc fractions produced small yet significant changes in IL-1α protein levels, with an increase observed for the EtOH fraction and a decrease observed for the EtOAc fraction (**Figure 5B**). Neither fraction resulted in substantial increases of IL-8 protein under basal conditions (<30 pg/mL; **Supplementary Figure 2**).

MAPK binding affinity results (**Figure 5C**) indicate additional potential targets that may support the immune response observed in RNA-Seq and ELISA results. MAPK binding affinity screening focused on the EtOAc agarikon fraction, which is commonly used for enrichment of semi-polar secondary metabolites in target-based binding assays. These assays indicated moderate and concentration-dependent binding activity toward JNK3 and TRKA, with percent control values decreasing from approximately 75% to 50% across the concentration range, consistent with increased ligand displacement.

### 3.5 Influenza A antiviral activity

**Figure 6:**
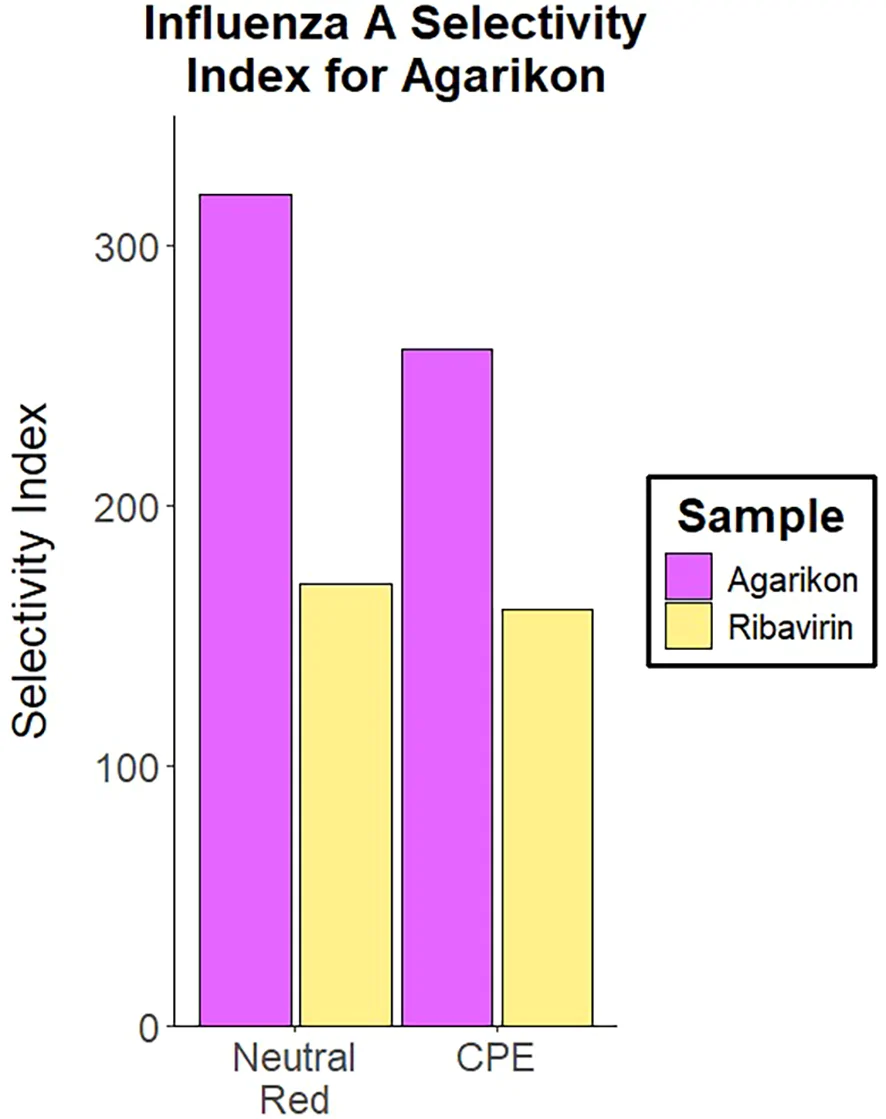
Selectivity index (SI) for agarikon mycelium against influenza A (H3N2) compared to ribavirin in neutral red and cytopathic effect (CPE) antiviral assays.

The antiviral activity of agarikon mycelium was of interest due to the consistently demonstrated antiviral activity of agarikon extracts, as well as the repeated presence of viral-related KEGG pathway results in the current study, including influenza A, Kaposi sarcoma-associated herpesvirus infection, tuberculosis, Epstein-Barr virus infection, hepatitis B, human T-cell leukemia virus 1 infection, and measles (*p*adj < 0.05). This specific strain of agarikon was previously included in a panel of polypore species extracts submitted for screening against numerous viruses as part of Project Bioshield, demonstrating especially notable activity against influenza A H3N2 (12; **Figure 6**). The selectivity index (SI) of a medicinal compound represents the ratio of its toxic concentration to its bioactive concentration, with values greater than 10 indicating a compound effective at a low concentration with a high concentration required for toxic effect, and values above 100 indicating notable therapeutic efficacy (29). As evaluated in neutral red and cytopathic effect assays, agarikon mycelium treatment demonstrated SI values of 260 or greater, up to 190% of those calculated for the antiviral drug ribavirin and far above the level accepted as both bioactive and safe.

## 4 Discussion

### 4.1 Immune response to agarikon treatment under basal conditions

Under basal conditions, agarikon treatment of human PBMCs supported a balanced immune response characterized by concurrent engagement of immune-activating pathways alongside anti-inflammatory and antioxidant signatures, as well as antiviral and antimicrobial immune cascades. *TLR4* (FC = -1.68) and *TLR7* (FC = -2.85) were selectively downregulated alongside upregulation of other innate immune effectors, as reflected in KEGG and Reactome pathway enrichment (**Figures 3A, C**). Although fungal β-glucans are TLR agonists, the downregulation of TLR transcripts and the TLR co-receptor *CD14* (FC = -1.72) paired with upregulation of negative regulators such as IL-1 receptor-associated kinase 3 (*IRAK3*; FC = 1.64) suggests that this immune response is prompted by other fungal components (30–33). Furthermore, this measured response includes the expression of a variety of complement cascade transcripts. While positive regulators such as complement component 1S (*C1S*; FC = 12.18) and properdin (*CFP*; FC = 1.71) are both engaged, the pathway is downregulated at many other stages of the complement cascade (complement C1q C chain (*C1QC*), FC = -1.42; complement C3a receptor 1 (*C3AR1*), FC = -1.43; complement factor D (*CFD*), FC = -1.99). Because *C3AR1* stimulus coactivates several TLRs, its downregulation may contribute to the notable *TLR4* and *TLR7* suppression observed (34, 35).

Agarikon treatment also resulted in modulation of cytokine signaling. *IL1RN*/*IL1RA* (FC = 6.94), which encodes IL-1RA protein, was even more upregulated than *IL1B* (FC = 5.09) and two separate agarikon preparations also induced significant IL-1RA protein production (**Figure 5A**). This pattern is consistent with regulatory modulation of the IL-1 axis rather than direct pro-inflammatory stimulation and aligns with immunomodulatory mechanisms proposed for agarikon and *T. versicolor* mycelium treatment in adjunctive COVID-19 vaccine contexts (8, 36). A disconnect between transcript and protein levels was observed for *IL8*, where robust transcriptional upregulation was not matched by comparable increases in secreted protein (**Supplementary Figure 2**). This pattern is consistent with a state of immune readiness or priming, in which cytokine-encoding transcripts are maintained at elevated levels without immediate translation into protein, enabling a more rapid response upon subsequent activation rather than reflecting active pro-inflammatory signaling at baseline (37–39). Similarly, IL-2 displayed divergent subunit expression (IL-2 receptor subunit gamma (*IL2RG*), FC = 1.28; IL-2 receptor subunit beta (*IL2RB*), FC = -1.20) paired with evidence of anti-inflammatory signals supported by IL-4 pathway modulation and significant *IL4I1* (FC = 4.37) upregulation (40). Taken together with T helper 2 cell (Th2) and IL-10-related pathway results, anti-inflammatory Th2 cell differentiation is likely, as IL-4 regulates the positive feedback loop for Th2 differentiation while also being produced by them, and IL-10 drives Th2 differentiation through regulation of dendritic cells (41). The strong engagement of these cascades and individual immune actors such as C-type lectin domain family 4 member E (*CLEC4E*/Mincle, FC = 4.97), which also positively regulates Th2 differentiation, supports a broader immunoregulatory profile associated with agarikon treatment (42–44). The noncanonical NF-κB pathway is known to be involved in positive regulation of anti-inflammatory Th2 cells (45), and agarikon treatment seems to modulate the NF-κB pathway, increasing transcript expression for subunits of both the canonical (*NFκB1*, FC = 1.29) and noncanonical (*NFKB2*, FC = 1.99; *RELB*, FC = 1.94) NF-κB pathways, alongside several NF-κB inhibitors (*NFκBIA*, FC = 2.39; NF-κB inhibitor epsilon (*NFκBIE*), FC = 1.88; NF-κB inhibitor zeta (*NFκBIZ*), FC = 1.39). These results are aligned with past reports of the anticancer effects of agarikon fruit body fractions driven by their upregulation of NF-κB proteins, including *NFKB1*/p50 and *NFKBIA*/IκBα, supporting the capacity of agarikon mycelium to provide a similar mechanistic drive towards immune modulation (13–15).

Transcripts engaged by agarikon mycelium treatment are largely involved with attenuation of inflammatory responses and redox modulation, either through direct antioxidant activity or negative regulation of inflammatory cellular markers. Antioxidant activity is reflected transcriptionally through the wide variety of antioxidant-related pathways and enzymes engaged such as the central regulator of mitochondrial redox balance *SOD2* (FC = 2.93), as well as glutathione peroxidase 4 (*GPX4*, FC = 1.46), peroxiredoxin 1 (*PRDX1*, FC = 1.36), thioredoxin (*TXN*, FC = 2.25), *TXNRD1* (FC = 1.39), and antioxidant 1 copper chaperone (*ATOX1*, FC = 1.85; 4, 13). Thioredoxin-interacting protein (*TXNIP*, FC = -1.44), the *TXN* inhibitor, is also downregulated. Additionally, several iron recycling pathway components are notably upregulated (six-transmembrane epithelial antigen of the prostate 4 (*STEAP4*), FC = 27.35; *STEAP3*, FC = 3.06; transferrin receptor (*TFRC*), FC = 2.26; cytochrome B561 family member A3 (*CYB561A3*), FC = 2.60) alongside family with sequence similarity 20, member C (*FAM20C*, FC = 2.28), which helps maintain ER redox homeostasis (46–48). Upregulation of these transcripts is likely responsible for the ferroptosis pathway response observed (**Figures 3A, C**), due to its reactive oxygen species (ROS)-based mechanism of action (49). This is further supported by the lack of cytotoxicity observed across a range of agarikon treatment concentrations and preparations, in conjunction with demonstration of its antioxidant ability and iron chelation potential (**Figures 1**, **4**). Although the functional consequences of agarikon binding to JNK3 remain to be determined, this interaction (**Figure 5C**) may be relevant to the coordinated redox remodeling observed in the present study, as JNK signaling has been implicated in oxidative stress responses and ferroptosis-associated cell death (50, 51).

Agarikon mycelium influenced a range of innate immune pathways spanning myeloid, dendritic, and cytokine signaling. For example, leukocyte immunoglobulin-like receptor subfamily B member 4 (*LILRB4*, FC = 1.77) helps inhibit myeloid cytokine production, while phospholipase A2 group VII (*PLA2G7*, FC = 3.62) inactivates inflammatory signaling molecules and decreases monocyte activity (52–54), similarly to *IL18BP* (FC = 2.25), which inhibits inflammatory IL-18 and the downstream production of IFN-γ (55). Secreted protein acidic and cysteine rich (*SPARC*, FC = 5.18) interacts with thrombospondin-1 (*THBS1*, FC = 7.17), which then regulates activity of a wide array of ligands; while the THBS1 interactome is complex, it is known to negatively regulate dendritic activation and can play an anti-inflammatory role (56, 57). Notably, transmembrane protein 176A (TMEM176A, FC = 3.16) is associated with immature dendritic cells, a hallmark of active immunomodulation or a lack of inflammatory signals as immature dendritic cells release much lower levels of inflammatory cytokines and T cell activators compared to mature cells (58, 59). Overall, while immunoactivating transcripts are upregulated by agarikon mycelial treatment under basal conditions, the accompanying engagement of regulatory and anti-inflammatory actors seems to pair with its demonstrated antioxidant activity to buffer the immune system away from extreme responses while at rest.

### 4.2 Immune modulation of agarikon treatment after LPS stimulation

Overall, the general pattern of immune engagement alongside simultaneous anti-inflammatory action was also observed under inflammatory conditions, though with additional (and appropriate) stimulation of immune effectors in response to LPS challenge. First, pathway results repeatedly implicate cytokine and interleukin signaling, and key messengers (*IL1A*, FC = 2.56; *IL1B*, FC = 2.01; *IL8*, FC = 3.14; *TLR5*, FC = 4.28) are significantly upregulated, indicating an appropriate response to LPS stimulation, where robust but regulated activation of these pathways is required for effective immune function. Interestingly, differences in extraction method resulted in a divergent IL-1α protein response, where the EtOH agarikon fraction resulted in small (<50 pg/mL) but significant increases in IL-1α, while the EtOAc fraction resulted in similarly significant decreases in IL-1α protein levels (**Figure 5B**). Overall, IL-1α protein concentrations under inflammatory conditions remained approximately two orders of magnitude lower than IL-1RA concentrations under basal conditions. Because IL-1RA competitively inhibits IL-1α signaling, these substantially higher IL-1RA levels align well with the reported 5- to 100-fold excess of IL-1RA needed to effectively antagonize IL-1α activity (60, 61).

Cytokine transcript upregulation is paired with the downregulation of three suppressors of cytokine signaling transcripts (*SOCS1*, FC = -1.53; *SOCS2*, FC = -2.18, *SOCS3*, FC = -1.65), key regulators of cytokine signaling feedback inhibition (62, 63). The inflammatory IL-18 axis is also suppressed, with downregulation of several IL-18 receptor components (IL-18 receptor 1 (*IL18R*), FC = -1.45; IL-18 receptor accessory protein (*IL18RAP*), FC = -1.51) and *IL18* (FC = -1.84) itself (64). This early response is also associated with the canonical NF-κB pathway, as it is positively regulated by TLR5 and IL-8 signaling (65). Additionally, both subunits of the noncanonical NF-κB pathway (*RELB*, FC = -1.28; *NFKB2*, FC = -1.19) are moderately repressed, while C-type lectin domain family 7 member A (*CLEC7A*/Dectin-1, FC = 4.71), a fungal β-glucan receptor, only appears as an upregulated transcript under inflammatory conditions. Dectin-1 is a well-established activator of the canonical NF-κB pathway through complexing with TLRs, which aligns with past observations of TLR and NF-κB engagement after agarikon treatment of cancer cells (13–15, 66–69). Notably, TLR and NF-κB subunit transcript expression exhibited opposing patterns under basal and inflammatory conditions, with reduced *TLR* and increased NF-κB subunit transcript expression at baseline reversing following LPS stimulation. Together, these findings suggest a context-dependent immunomodulatory response.

In contrast to the basal response, agarikon treatment under LPS stimulation seems to have prompted complement cascade activation (complement C3 (*C3*), FC = 3.21; complement C5a receptor 1 (*C5AR1*), FC = 3.59; complement C5a receptor 2 (*C5AR2*), FC = 3.81), indicating an enhanced engagement of innate immune effector mechanisms. However, the C-type lectins expressed reflected a balance between immune activation and immune regulation. While there was notable upregulation of *CLEC10A* (FC = 173.84), which promotes IL-10 production by dendritic cells, its induction was coupled with *CLEC5A* (FC = 10.98), a myeloid innate immune checkpoint that participates in pro-inflammatory signaling (70, 71). Lectin modulation was also evident among galectins involved in innate and adaptive immune regulation, including galectin-2 (*LGALS2*, FC = 2.46) and *LGALS3* (FC = 2.24). *LGALS3* has complex signaling effects, helping to upregulate inflammatory signals via the NLR family pyrin domain containing 3 (NLRP3) inflammasome, IL-1β production, and neutrophil recruitment, while also preventing over-engagement by raising the threshold for T cell activation and positively regulating anti-inflammatory M2 macrophages (72–78). This may suggest a role for galectin signaling in agarikon-mediated modulation of the IL-1 axis.

While a robust immune response to challenge is desirable, excessive inflammation can be detrimental. Agarikon treatment appears to maintain this signaling balance through influencing a wide variety of immune signaling networks to aid cellular response to LPS. For example, macrophage-expressed 1 (*MPEG1*, FC = 1.79) is an antimicrobial pore-forming protein upregulated by LPS, and stabilin-1 (*STAB1*, FC = 9.45) is a scavenger receptor that helps to clear LPS from the bloodstream (79, 80). This response was also characterized by modest downregulation of seven aminoacyl-tRNA synthetase transcripts (ARSs), including multiple cytosolic isoforms, which play roles beyond protein translation in inflammatory and oncogenic signaling (81–83). A small set of transcripts associated with inflammatory activation and poor cancer prognosis, including interferon induced transmembrane protein 1 (*IFITM1*, FC = −1.21), adhesion G protein-coupled receptor E5 (*ADGRE5*, FC = −1.30), and *CCL19* (FC = −13.07), were also reduced, consistent with attenuation of inflammatory amplification processes (84–87). Conversely, expression of *FUCA1* (FC = 4.12) is associated with better cancer survival rates and may play a direct suppressive role in tandem with galactose-3-O-sulfotransferase 4 (*GAL3ST4*, FC = 2.23), which promotes macrophage M2 transition (88–90). Additionally, transmembrane protein 119 (*TMEM119*, FC = 10.33) helps suppress microglial inflammatory reactions after strokes (91), and this strongly expressed candidate is notable in the context of agarikon’s demonstrated binding affinity for TRKA (**Figure 5C**), the primary receptor of nerve growth factor (NGF) in the brain.

Finally, agarikon-induced redox remodeling was maintained under LPS stimulation, indicating that antioxidant- and ferroptosis-related protective signatures persist despite inflammatory ROS generation. Pathway-level signals point to engagement of iron recycling and ROS detoxification processes linked to resolution of inflammatory oxidative stress, supporting maintenance of redox homeostasis during immune activation. This response was supported by the upregulation of glutathione-disulfide reductase (*GSR*, FC = 1.45), *TXN* (FC = 1.71), and *TXNRD1* (FC = 1.75), alongside ferroptosis suppressor solute carrier family 7 member 11 (*SLC7A11*, FC = 3.20) and NAD(P)H quinone dehydrogenase 1 (*NQO1*, FC = 2.29), a stress-inducible enzyme involved in cellular protection against redox imbalance (92–94). Overall, the immune engagement prompted by agarikon treatment of human PBMCs after LPS challenge seems to be tightly regulated by concurrent immune-pacifying and immune-engaging signals, with parallel maintenance of redox balance.

### 4.3 Translational relevance of agarikon immunomodulation

**Figure 7:**
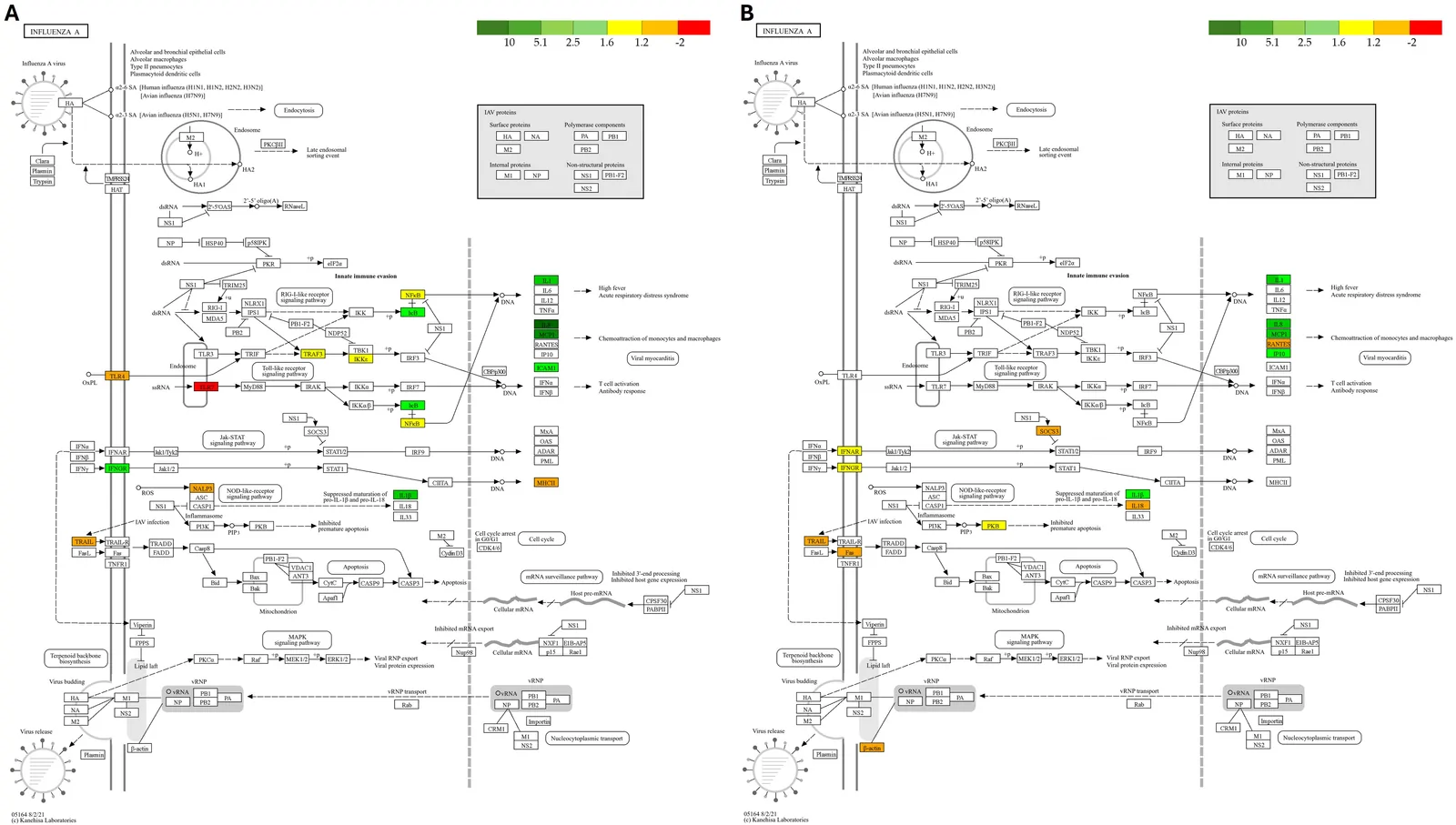
KEGG pathway for influenza A with genes affected by agarikon treatment under **(A)** basal and **(B)** inflammatory conditions highlighted according to average FC (n = 4, DESeq2, *p*adj < 0.05).

The transcriptomic immunomodulatory profile showed a clear overlap with antiviral-associated pathways, supporting a potential mechanistic basis for the well-established antiviral activity of agarikon observed in functional and clinical contexts (9, 10, 12, 18). Agarikon treatment engaged key mediators known to support innate immune activation and viral clearance, such as *IL8* and *IL1B* (**Figures 3G**, **7A, B**), while also inducing significant virus-related pathway results (**Figures 3A–D**), notably including influenza A (95–97). These system-level insights align with functional antiviral profiling of agarikon mycelial extract against influenza A (H3N2) infection (**Figure 6**), which demonstrated SI values exceeding 300 in some assays and consistently higher activity than ribavirin alongside low cytotoxicity. Influenza-related antiviral activity of agarikon has also been reported in independent studies, including a prior investigation of the antiviral activity of aqueous extracts of agarikon mycelium against H5N1 and H3N3, which produced significant reductions in viral titers at concentrations that remained non-cytotoxic (18). Furthermore, the strain of agarikon mycelium employed in the current study has been evaluated clinically in combination with *T. versicolor* mycelium in multiple contexts, using unfermented rice as a placebo control. One clinical trial (9) investigating the use of this fungal preparation as a COVID-19 treatment has established that this agarikon and *T. versicolor* mycelium blend is a safe and well-tolerated intervention associated with reduced number and severity of COVID-19 symptoms, with supportive preclinical findings demonstrating significant reductions in viral entry in mammalian epithelial cells (Vero-TMPRSS2). Agarikon and *T. versicolor* mycelium also show promise as a safe COVID-19 vaccine adjunct, where the blend was associated with reduced vaccine-related side effects without compromising SARS-CoV-2 antibody responses, and potential indications of increased antibody levels for at least six months in patients without prior COVID-19 exposure (8).

In addition to its inclusion in traditional European medicinal preparations termed “elixir[s] of long life,” modern research suggests a potential role for agarikon in supporting longevity-related biological processes, particularly under stress conditions (98, 99). The same properties that contribute to the antiviral and antimicrobial action of agarikon raise the possibility that this species could ameliorate age-related diseases (ARDs) driven by long-term inflammation (2, 4, 6, 10, 12, 14, 15). These findings may also have relevance to healthy ageing, as immune dysregulation and redox imbalance are central features of ARDs and inflammaging, the long-term, low-grade inflammation induced by chronic stimulation of the innate immune system (100–103). The coordinated antioxidant and context-dependent immunomodulatory responses observed here are consistent with mechanisms associated with resilience to chronic inflammatory stress, although these implications would benefit from clinical validation (104–108). The antioxidant profile supported by agarikon aligns with findings from a D-galactose-induced mouse ageing model, in which agarikon improved antioxidant enzyme activities (SOD, CAT, and GSH-Px), reduced malondialdehyde (MDA) levels, and improved tissue indices associated with ageing (99). Collectively, these findings support a role for agarikon mycelium in regulating host responses across multiple stress contexts, characterized by immune buffering and redox homeostasis.

This study has several limitations. Although selected cytokines were validated at the protein level, many of the immunomodulatory pathways identified by transcriptomic analysis have not yet been mechanistically confirmed. Receptor-blocking or pathway-specific inhibition studies would help establish which signaling pathways are causally responsible for the observed effects. The differential, and potentially complementary, immunomodulatory activity observed among agarikon extracts also highlights the importance of extraction methodology in determining biological activity. More comprehensive chemical characterization and bioactivity-guided fractionation will be important for identifying the constituents responsible for these effects. Finally, while these findings provide a preclinical foundation for the immunomodulatory potential of cultivated agarikon, *in vivo* and human clinical studies are needed to establish their translational relevance.

## 5 Conclusion

Despite millennia of traditional use and modern clinical application, agarikon treatment remains incompletely characterized with respect to mechanistic immune outcomes. Building on findings reported for the fruit body, this work highlights coordinated immunoregulatory effects elicited by agarikon mycelium, providing context for preclinical studies and clinical investigations of individual and blended preparations including agarikon and supporting hypotheses regarding its specifics of action, such as IL-1 axis modulation. While mycelial and fruit body preparations seem to produce both overlapping and distinct biological effects, this study further supports the immune-buffering capacity of mycelium-based treatment and highlights overlap with fruit body findings, including engagement of NF-κB signaling. Given the endangered status of agarikon, mycelial cultivation offers a scalable and sustainable approach to accessing its bioactive potential, underscoring the importance of continued research on this material. Overall, these findings support a role for agarikon in promoting adaptive resilience under stressed conditions, characterized by coordinated immunomodulatory activity, antiviral activity, and maintenance of redox balance.

## Data Availability

The data presented in the study are deposited in the NCBI repository, Bioproject number PRJNA1505714. Further inquiries can be directed to the corresponding author.

## References

[1] Dioscorides Pedanius Osbaldeston TA Wood RP . De Materia Medica: Being an Herbal With Many Other Medicinal Materials: Written in Greek in the First Century of the Common Era: A New Indexed Version in Modern English. Johannesburg: IBIDIS (2000).

[2] Gafforov Y Rašeta M Mykchaylova O Rapior S Karaman M Mišković J . Mycochemistry, traditional uses, and nutraceutical potential of *Laricifomes officinalis*: A biotechnological and pharmacological perspective. Plant Foods Hum Nutr. (2025) 80:77. doi: 10.1007/s11130-025-01316-9 39988639

[3] Tayjanov K Khojimatov O Gafforov Y Makhkamov T Normakhamatov N Bussmann RW . Plants and fungi in the ethnomedicine of the medieval East - a review. Ethnobotany Res Appl. (2021) 22:1–20. doi: 10.32859/era.22.46.1-2041963607

[4] Fijałkowska A Muszyńska B Sułkowska-Ziaja K Kała K Pawlik A Stefaniuk D . Medicinal potential of mycelium and fruiting bodies of an arboreal mushroom *Fomitopsis officinalis* in therapy of lifestyle diseases. Sci Rep. (2020) 10:20081. doi: 10.1038/s41598-020-76899-1 33208786 PMC7674418

[5] Girometta C . Antimicrobial properties of *Fomitopsis officinalis* in the light of its bioactive metabolites: a review. Mycology. (2019) 10:32–9. doi: 10.1080/21501203.2018.1536680 30834150 PMC6394315

[6] Hwang CH Jaki BU Klein LL McAlpine JB Napolitano JG Fryling NA . Chlorinated coumarins from the polypore mushroom *Fomitopsis officinalis* and their activity against *Mycobacterium tuberculosis*. J Nat Prod. (2013) 76:1916–22. doi: 10.1021/np400497f 24087924 PMC3851412

[7] Karunarathna SC Patabendige NM Kumla J Hapuarachchi KK Suwannarach N . The bioactive compounds, beneficial medicinal properties, and biotechnological prospects of *Fomitopsis*: a comprehensive overview. Front Cell Infect Microbiol. (2025) 15. doi: 10.3389/fcimb.2025.1534617 40330023 PMC12053173

[8] Saxe G Smith CN Golshan S Shekhtman T Bair ZJ Beathard C . Polypore mushroom mycelia as an adjunct to COVID-19 vaccination: a randomized clinical trial. BMC Immunol. (2026) 27:24. doi: 10.1186/s12865-026-00809-9 41620671 PMC12955250

[9] Saxe G Shubov A Smith CN Golshan S Shekhtman T Wilson S . Polypore mushroom mycelia for treatment of active COVID-19 infection: A randomized clinical trial. medRxiv. (2026). doi: 10.64898/2026.06.01.26354267 PMC12955250 41620671

[10] Sidorenko ML Buzoleva LS . Search for new types of raw materials for antibacterial drugs. Antibiot Khimioter. (2012) 57:7–10 23156038.

[11] Stamets PE . Medicinal polypores of the forests of North America: Screening for novel antiviral activity. IJM. (2005) 7:362. doi: 10.1615/IntJMedMushrooms.v7.i3.21036976008

[12] Stamets P . Antipox properties of *Fomitopsis officinalis* (Vill.: Fr.) Bond. et Singer (agarikon) from the Pacific Northwest of North America. Int J Med Mushr. (2005) 7:495–506. doi: 10.1615/IntJMedMushr.v7.i3.6036976008

[13] Altannavch N Zhou X Khan MA Ahmed A Naranmandakh S Fu JJ . Anti-oxidant and anticancerous effect of *Fomitopsis officinalis* (Vill. ex Fr. Bond. et Sing) mushroom on hepatocellular carcinoma cells *in vitro* through NF-kB pathway. Anti-Cancer Agents Med Chem. (2022) 22:1561–70. doi: 10.2174/1871520621666210608101152 34102992

[14] Liu W Shen Y Hou J Jiang H Wang Q Zhang L . A fungal polysaccharide from *Fomitopsis officinalis* as a multi-target molecule to combat cancer. Int J Biol Macromol. (2024) 272:132543. doi: 10.1016/j.ijbiomac.2024.132543 38788870

[15] Shen Y Hou J Liu W Lin Z Ma L Xu J . An antitumor fungal polysaccharide from *Fomitopsis officinalis* by activating immunity and inhibiting angiogenesis. Int J Biol Macromol. (2024) 267:131320. doi: 10.1016/j.ijbiomac.2024.131320 38569989

[16] Davis R Taylor A Nally R Benson KF Stamets P Jensen GS . Differential immune activating, anti-inflammatory, and regenerative properties of the aqueous, ethanol, and solid fractions of a medicinal mushroom blend. J Inflammation Res. (2020) 13:117–31. doi: 10.2147/JIR.S229446 32158252 PMC7049272

[17] Doar E Kishiyama J Bair ZJ Beathard C . Calm under challenge: Immune-balancing and stress-quenching effects of *Hericium erinaceus* mycelium in human immune cells. Immuno. (2026) 6:2. doi: 10.3390/immuno601000230654563

[18] Teplyakova TV Psurtseva NV Kosogova TA Mazurkova NA Khanin VA Vlasenko VA . Antiviral activity of polyporoid mushrooms (higher Basidiomycetes) from Altai Mountains (Russia). IJM. (2012) 14:37–45. doi: 10.1615/IntJMedMushr.v14.i1.40 22339706

[19] Gill SK Rieder MJ . Toxicity of a traditional Chinese medicine, *Ganoderma lucidum*, in children with cancer. Can J Clin Pharmacol. (2008) 15:e275–85 18603664.

[20] Kałucka IL Svetasheva T . IUCN red list. In: IUCN Red List of Threatened Species: Fomitopsis Officinalis Cambridge: IUCN Biodiversity Assessment & Knowledge Team: Red List Unit (2019). doi: 10.2305/IUCN.UK.2019-3.RLTS.T75104087A75104095.en

[21] Zalesky T Bradshaw AJ Bair ZJ Meyer KW Stamets P . Fungal cryopreservation across 61 genera: Practical application and method evaluation. Mycologia. (2024) 116:865–76. doi: 10.1080/00275514.2024.2363135 38949868

[22] Bennett PI Bair ZJ Bradshaw AJ Stamets P . Genomic characterization of the endangered medicinal polypore agarikon (*Laricifomes officinalis* syn. *Fomitopsis officinalis*). G3. (2026) in press. doi: 10.1093/g3journal/jkag237 PMC13732445 42641138

[23] Ehlers SM Maxein J Koop JHE . Low-cost microplastic visualization in feeding experiments using an ultraviolet light-emitting flashlight. Ecol Res. (2020) 35:265–73. doi: 10.1111/1440-1703.1208040046247

[24] Love MI Huber W Anders S . Moderated estimation of fold change and dispersion for RNA-seq data with DESeq2. Genome Biol. (2014) 15:550. doi: 10.1186/s13059-014-0550-8 25516281 PMC4302049

[25] Fabian MA Biggs WH Treiber DK Atteridge CE Azimioara MD Benedetti MG . A small molecule–kinase interaction map for clinical kinase inhibitors. Nat Biotechnol. (2005) 23:329–36. doi: 10.1038/nbt1068 15711537

[26] Karaman MW Herrgard S Treiber DK Gallant P Atteridge CE Campbell BT . A quantitative analysis of kinase inhibitor selectivity. Nat Biotechnol. (2008) 26:127–32. doi: 10.1038/nbt1358 18183025

[27] Dinis TCP Madeira VMC Almeida LM . Action of phenolic derivatives (acetaminophen, salicylate, and 5-aminosalicylate) as inhibitors of membrane lipid peroxidation and as peroxyl radical scavengers. Arch Biochem Biophys. (1994) 315:161–9. doi: 10.1006/abbi.1994.1485 7979394

[28] Yusof N Hasan MH Armayni UA Ahmad MSB Wahab IA . The ferrous iron chelating assay of extracts. Open Conf Proc J. (2013) 4:1555. doi: 10.2174/2210289201304010015533718602

[29] Indrayanto G Satrio Putra G Suhud F . Validation of in-vitro bioassay methods: Application in herbal drug research, in: Profiles of Drug Substances, Excipients and Related Methodology (2021). Elsevier. Available online at: https://linkinghub.elsevier.com/retrieve/pii/S1871512520300170 (Accessed April 10, 2026). 10.1016/bs.podrm.2020.07.005 33461699

[30] Ghosh TK Mickelson DJ Solberg JC Lipson KE Inglefield JR Alkan SS . TLR-TLR cross talk in human PBMC resulting in synergistic and antagonistic regulation of type-1 and 2 interferons, IL-12 and TNF-alpha. Int Immunopharmacol. (2007) 7:1111–21. doi: 10.1016/j.intimp.2007.04.006 17570328

[31] Kanjan P Sahasrabudhe NM de Haan BJ de Vos P . Immune effects of β-glucan are determined by combined effects on dectin-1, TLR2, 4 and 5. J Funct Foods. (2017) 37:433–40. doi: 10.1016/j.jff.2017.07.06142574925

[32] Quayle K Coy C Standish L Lu H . The TLR2 agonist in polysaccharide-K is a structurally distinct lipid which acts synergistically with the protein-bound β-glucan. J Nat Med. (2015) 69:198–208. doi: 10.1007/s11418-014-0879-z 25510899

[33] Thiel FG Asgarbeik S Glaubitz J Wilden A Lerch MM Weiss FU . IRAK3-mediated suppression of pro-inflammatory MyD88/IRAK signaling affects disease severity in acute pancreatitis. Sci Rep. (2023) 13:10833. doi: 10.1038/s41598-023-37930-3 37402858 PMC10319718

[34] Merle NS Church SE Fremeaux-Bacchi V Roumenina LT . Complement system part I – molecular mechanisms of activation and regulation. Front Immunol. (2015) 6. doi: 10.3389/fimmu.2015.00262 26082779 PMC4451739

[35] Merle NS Noe R Halbwachs-Mecarelli L Fremeaux-Bacchi V Roumenina LT . Complement system part II: Role in immunity. Front Immunol. (2015) 6. doi: 10.3389/fimmu.2015.00257 26074922 PMC4443744

[36] Carter DB Deibel MR Dunn CJ Tomich CS Laborde AL Slightom JL . Purification, cloning, expression and biological characterization of an interleukin-1 receptor antagonist protein. Nature. (1990) 344:633–8. doi: 10.1038/344633a0 2139180

[37] Asada N Ginsberg P Paust HJ Song N Riedel JH Turner JE . The integrated stress response pathway controls cytokine production in tissue-resident memory CD4+ T cells. Nat Immunol. (2025) 26:557–66. doi: 10.1038/s41590-025-02105-x 40050432 PMC11957990

[38] Hoffmann E Dittrich-Breiholz O Holtmann H Kracht M . Multiple control of interleukin-8 gene expression. J Leukocyte Biol. (2002) 72:847–55. doi: 10.1189/jlb.72.5.847 12429706

[39] Vacchini A Mortier A Proost P Locati M Metzemaekers M Borroni EM . Differential effects of posttranslational modifications of CXCL8/interleukin-8 on CXCR1 and CXCR2 internalization and signaling properties. Int J Mol Sci. (2018) 19:3768. doi: 10.3390/ijms19123768 30486423 PMC6321254

[40] Romagnani S . IL4I1: Key immunoregulator at a crossroads of divergent T-cell functions. Eur J Immunol. (2016) 46:2302–5. doi: 10.1002/eji.201646617 27726138

[41] Zhang Y Zhang Y Gu W Sun B . Th1/th2 cell differentiation and molecular signals, in: T Helper Cell Differentiation and Their Function (2014). Dordrecht: Springer Netherlands. doi: 10.1007/978-94-017-9487-9_2 (Accessed July 24, 2025).

[42] Liu L Rich BE Inobe J Chen W Weiner HL . Induction of Th2 cell differentiation in the primary immune response: dendritic cells isolated from adherent cell culture treated with IL-10 prime naive CD4+ T cells to secrete IL-4. Int Immunol. (1998) 10:1017–26. doi: 10.1093/intimm/10.8.1017 9723687

[43] Wevers BA . C-type lectin signaling in dendritic cells: molecular control of antifungal inflammation (2014). Available online at: https://dare.uva.nl/search?identifier=6719dc68-07a7-4e98-b598-1194d0320c05 (Accessed July 10, 2025).

[44] Wevers BA Kaptein TM Zijlstra-Willems EM Theelen B Boekhout T Geijtenbeek TBH . Fungal engagement of the C-type lectin mincle suppresses dectin-1-induced antifungal immunity. Cell Host Microbe. (2014) 15:494–505. doi: 10.1016/j.chom.2014.03.008 24721577

[45] Oh H Ghosh S . NF-κB: roles and regulation in different CD4+ T-cell subsets. Immunol Rev. (2013) 252:41–51. doi: 10.1111/imr.12033 23405894 PMC3576882

[46] Meng F Fleming BA Jia X Rousek AA Mulvey MA Ward DM . Lysosomal iron recycling in mouse macrophages is dependent upon both LcytB and Steap3 reductases. Blood Adv. (2022) 6:1692–707. doi: 10.1182/bloodadvances.2021005609 34982827 PMC8941456

[47] Zhang J Zhu Q Wang X Yu J Chen X Wang J . Secretory kinase Fam20C tunes endoplasmic reticulum redox state via phosphorylation of Ero1α. EMBO J. (2018) 37:e98699. doi: 10.15252/embj.201798699 29858230 PMC6043849

[48] Zhou X An Z Lei H Liao H Guo X . Role of the human cytochrome b561 family in iron metabolism and tumors (Review). Oncol Lett. (2025) 29:111. doi: 10.3892/ol.2024.14857 39802312 PMC11718626

[49] Li J Cao F Yin H Huang Z Lin Z Mao N . Ferroptosis: past, present and future. Cell Death Dis. (2020) 11:1–13. doi: 10.1038/s41419-020-2298-2 32015325 PMC6997353

[50] Cui J Ma Q Zhang C Li Y Liu J Xie K . Keratin 18 depletion as a possible mechanism for the induction of apoptosis and ferroptosis in the rat hippocampus after hypobaric hypoxia. Neuroscience. (2023) 513:64–75. doi: 10.1016/j.neuroscience.2022.11.009 36395917

[51] Zheng X Liang Y Zhang C . Ferroptosis regulated by hypoxia in cells. Cells. (2023) 12:1050. doi: 10.3390/cells12071050 37048123 PMC10093394

[52] Tew DG Southan C Rice SQ Lawrence MP Li H Boyd HF . Purification, properties, sequencing, and cloning of a lipoprotein-associated, serine-dependent phospholipase involved in the oxidative modification of low-density lipoproteins. Arterioscler Thromb Vasc Biol. (1996) 16:591–9. doi: 10.1161/01.atv.16.4.591 8624782

[53] Tjoelker LW Wilder C Eberhardt C Stafforini DM Dietsch G Schimpf B . Anti-inflammatory properties of a platelet-activating factor acetylhydrolase. Nature. (1995) 374:549–53. doi: 10.1038/374549a0 7700381

[54] Yang T Qian Y Liang X Wu J Zou M Deng M . LILRB4, an immune checkpoint on myeloid cells. Blood Sci. (2022) 4:49–56. doi: 10.1097/BS9.0000000000000109 35957669 PMC9362873

[55] Yasuda K Nakanishi K Tsutsui H . Interleukin-18 in health and disease. Int J Mol Sci. (2019) 20:3. doi: 10.3390/ijms20030649 30717382 PMC6387150

[56] Doyen V Rubio M Braun D Nakajima T Abe J Saito H . Thrombospondin 1 is an autocrine negative regulator of human dendritic cell activation. J Exp Med. (2003) 198:1277–83. doi: 10.1084/jem.20030705 14568985 PMC2194231

[57] Resovi A Pinessi D Chiorino G Taraboletti G . Current understanding of the thrombospondin-1 interactome. Matrix Biol. (2014) Matricellular Proteins37:83–91. doi: 10.1016/j.matbio.2014.01.012 24476925

[58] Condamine T Le Texier L Howie D Lavault A Hill M Halary F . Tmem176B and Tmem176A are associated with the immature state of dendritic cells. J Leukoc Biol. (2010) 88:507–15. doi: 10.1189/jlb.1109738 20501748

[59] Coombes JL Powrie F . Dendritic cells in intestinal immune regulation. Nat Rev Immunol. (2008) 8:435–46. doi: 10.1038/nri2335 18500229 PMC2674208

[60] Arend WP Welgus HG Thompson RC Eisenberg SP . Biological properties of recombinant human monocyte-derived interleukin 1 receptor antagonist. J Clin Invest. (1990) 85:1694–7. doi: 10.1172/JCI114622 2139669 PMC296623

[61] Palomo J Dietrich D Martin P Palmer G Gabay C . The interleukin (IL)-1 cytokine family--Balance between agonists and antagonists in inflammatory diseases. Cytokine. (2015) 76:25–37. doi: 10.1016/j.cyto.2015.06.017 26185894

[62] Fujimoto M Naka T . Regulation of cytokine signaling by SOCS family molecules. Trends Immunol. (2003) 24:659–66. doi: 10.1016/j.it.2003.10.008 14644140

[63] Krebs DL Hilton DJ . SOCS: physiological suppressors of cytokine signaling. J Cell Sci. (2000) 113:2813–9. doi: 10.1242/jcs.113.16.2813 10910765

[64] Dinarello CA Novick D Kim S Kaplanski G . Interleukin-18 and IL-18 binding protein. Front Immunol. (2013) 4:289. doi: 10.3389/fimmu.2013.00289 24115947 PMC3792554

[65] Yu Y Zeng H Lyons S Carlson A Merlin D Neish AS . TLR5-mediated activation of p38 MAPK regulates epithelial IL-8 expression via posttranscriptional mechanism. Am J Physiology-Gastrointestinal Liver Physiol. (2003) 285:G282–90. doi: 10.1152/ajpgi.00503.2002 12702497

[66] Ferwerda G Meyer-Wentrup F Kullberg BJ Netea MG Adema GJ . Dectin-1 synergizes with TLR2 and TLR4 for cytokine production in human primary monocytes and macrophages. Cell Microbiol. (2008) 10:2058–66. doi: 10.1111/j.1462-5822.2008.01188.x 18549457

[67] Gantner BN Simmons RM Canavera SJ Akira S Underhill DM . Collaborative induction of inflammatory responses by dectin-1 and Toll-like receptor 2. J Exp Med. (2003) 197:1107–17. doi: 10.1084/jem.20021787 12719479 PMC2193968

[68] Roebuck KA . Oxidant stress regulation of IL-8 and ICAM-1 gene expression: differential activation and binding of the transcription factors AP-1 and NF-kappaB (Review). Int J Mol Med. (1999) 4:223–53. doi: 10.3892/ijmm.4.3.223 10425270

[69] Shen L Xiao Y Wu Q Liu L Zhang C Pan X . TLR4/NF-κB axis signaling pathway-dependent up-regulation of miR-625-5p contributes to human intervertebral disc degeneration by targeting COL1A1. Am J Transl Res. (2019) 11:1374–88. 30972168 PMC6456508

[70] Hoober JK . ASGR1 and its enigmatic relative, CLEC10A. Int J Mol Sci. (2020) 21:4818. doi: 10.3390/ijms21144818 32650396 PMC7404283

[71] Tosiek MJ Groesser K Pekcec A Zwirek M Murugesan G Borges E . Activation of the innate immune checkpoint CLEC5A on myeloid cells in the absence of danger signals modulates macrophages’ function but does not trigger the adaptive T cell immune response. J Immunol Res. (2022) 2022:9926305. doi: 10.1155/2022/9926305 35252461 PMC8896916

[72] Beccaria CG Amezcua Vesely MC Fiocca Vernengo F Gehrau RC Ramello MC Tosello Boari J . Galectin-3 deficiency drives lupus-like disease by promoting spontaneous germinal centers formation via IFN-γ. Nat Commun. (2018) 9:1628. doi: 10.1038/s41467-018-04063-5 29691398 PMC5915532

[73] Bhaumik P St-Pierre G Milot V St-Pierre C Sato S . Galectin-3 facilitates neutrophil recruitment as an innate immune response to a parasitic protozoa cutaneous infection. J Immunol. (2013) 190:630–40. doi: 10.4049/jimmunol.1103197 23241887

[74] Chen HY Fermin A Vardhana S Weng IC Lo KFR Chang EY . Galectin-3 negatively regulates TCR-mediated CD4+ T-cell activation at the immunological synapse. Proc Natl Acad Sci. (2009) 106:14496–501. doi: 10.1073/pnas.0903497106 19706535 PMC2732795

[75] Liu FT Stowell SR . The role of galectins in immunity and infection. Nat Rev Immunol. (2023) 23:1–16. doi: 10.1038/s41577-022-00829-7 36646848 PMC9842223

[76] MacKinnon AC Farnworth SL Hodkinson PS Henderson NC Atkinson KM Leffler H . Regulation of alternative macrophage activation by galectin-3. J Immunol. (2008) 180:2650–8. doi: 10.4049/jimmunol.180.4.2650 18250477

[77] Sano H Hsu DK Apgar JR Yu L Sharma BB Kuwabara I . Critical role of galectin-3 in pagocytosis by macrophages. J Clin Invest. (2003) 112:389–97. doi: 10.1172/JCI17592 12897206 PMC166291

[78] Tian J Yang G Chen HY Hsu DK Tomilov A Olson KA . Galectin-3 regulates inflammasome activation in cholestatic liver injury. FASEB J. (2016) 30:4202–13. doi: 10.1096/fj.201600392RR 27630169 PMC5102125

[79] Bayly-Jones C Pang SS Spicer BA Whisstock JC Dunstone MA . Ancient but not forgotten: New insights into MPEG1, a macrophage perforin-like immune effector. Front Immunol. (2020) 11. doi: 10.3389/fimmu.2020.581906 33178209 PMC7593815

[80] Cabral F Al-Rahem M Skaggs J Thomas TA Kumar N Wu Q . Stabilin receptors clear LPS and control systemic inflammation. iScience. (2021) 24. doi: 10.1016/j.isci.2021.103337 34816100 PMC8591421

[81] Pang YLJ Poruri K Martinis SA . tRNA synthetase: tRNA aminoacylation and beyond. WIREs RNA. (2014) 5:461–80. doi: 10.1002/wrna.1224 24706556 PMC4062602

[82] Park SG Schimmel P Kim S . Aminoacyl tRNA synthetases and their connections to disease. Proc Natl Acad Sci USA. (2008) 105:11043–9. doi: 10.1073/pnas.0802862105 18682559 PMC2516211

[83] Sangha AK Kantidakis T . The aminoacyl-tRNA synthetase and tRNA expression levels are deregulated in cancer and correlate independently with patient survival. Curr Issues Mol Biol. (2022) 44:7. doi: 10.3390/cimb44070207 35877431 PMC9324904

[84] Gowhari Shabgah A Al-Obaidi ZMJ Sulaiman Rahman H Kamal Abdelbasset W Suksatan W Bokov DO . Does CCL19 act as a double-edged sword in cancer development? Clin Exp Immunol. (2022) 207:164–75. doi: 10.1093/cei/uxab039 35020885 PMC8982982

[85] Hsiao CC Keysselt K Chen HY Sittig D Hamann J Lin HH . The adhesion GPCR CD97/ADGRE5 inhibits apoptosis. Int J Biochem Cell Biol. (2015) 65:197–207. doi: 10.1016/j.biocel.2015.06.007 26071181

[86] Zhang L Shi L . The E2F1/MELTF axis fosters the progression of lung adenocarcinoma by regulating the Notch signaling pathway. Mutat Res Fundam Mol Mech Mutagen. (2023) 827:111837. doi: 10.1016/j.mrfmmm.2023.111837 37820570

[87] Zhang X Zhang X Yang Q Han R Fadhul W Sachdeva A . Comprehensive analysis of ADGRE5 gene in human tumors: Clinical relevance, prognostic implications, and potential for personalized immunotherapy. Heliyon. (2024) 10. doi: 10.1016/j.heliyon.2024.e27459 38501000 PMC10945187

[88] Liu H Zhang C Peng S Yin Y Xu Y Wu S . Prognostic models of immune-related cell death and stress unveil mechanisms driving macrophage phenotypic evolution in colorectal cancer. J Transl Med. (2025) 23:127. doi: 10.1186/s12967-025-06143-9 39875913 PMC11776142

[89] Shi L Yuan D Zhu F He Y Zuo R Chen L . Immune-related gene risk model establishment and role of key gene FUCA1 in Malignant pleural mesothelioma. Front Pharmacol. (2025) 16. doi: 10.3389/fphar.2025.1577232 40487392 PMC12141270

[90] Wang X Dou J Liu M Zhang Y Li Y Tong Z . Potential predictive value of immune-related genes FUCA1 and NCKAP1L for osteosarcoma metastasis. Gene. (2024) 927:148645. doi: 10.1016/j.gene.2024.148645 38844271

[91] Ngwa C Sharmeen R Xu Y Li J McCullough LD Liu F . Microglial Tmem119 inhibits IRAK4 activation and confers neuroprotection after ischemic stroke. Stroke. (2024) 55:AWP312–2. doi: 10.1161/str.55.suppl_1.WP312

[92] Dinkova-Kostova AT Talalay P . NAD(P)H:quinone acceptor oxidoreductase 1 (NQO1), a multifunctional antioxidant enzyme and exceptionally versatile cytoprotector. Arch Biochem Biophys. (2010) 501:116–23. doi: 10.1016/j.abb.2010.03.019 20361926 PMC2930038

[93] Fiore A Zeitler L Russier M Groß A Hiller MK Parker JL . Kynurenine importation by SLC7A11 propagates anti-ferroptotic signaling. Mol Cell. (2022) 82:920–932.e7. doi: 10.1016/j.molcel.2022.02.007 35245456 PMC7617904

[94] Li Z Xu X Leng X He M Wang J Cheng S . Roles of reactive oxygen species in cell signaling pathways and immune responses to viral infections. Arch Virol. (2017) 162:603–10. doi: 10.1007/s00705-016-3130-2 27848013

[95] Ander SE Li FS Carpentier KS Morrison TE . Innate immune surveillance of the circulation: A review on the removal of circulating virions from the bloodstream. PloS Pathog. (2022) 18:e1010474. doi: 10.1371/journal.ppat.1010474 35511797 PMC9070959

[96] Mukherjee S Dutta S Pati S Sarkar T Paul S Chakraborty S . Nuclear factor kappa-B: The El Dorado of inflammatory immune response. J Cell Mol Immunol. (2024) 3:Issue 1. doi: 10.46439/immunol.3.025

[97] Newton AH Cardani A Braciale TJ . The host immune response in respiratory virus infection: balancing virus clearance and immunopathology. Semin Immunopathol. (2016) 38:471–82. doi: 10.1007/s00281-016-0558-0 26965109 PMC4896975

[98] Schultz F Niedenthal T Nicolai-Lorenz I Baez K Iker T Garbe LA . A potion for prolonged life? Germes’ recipe, a secret handwritten note from an 18th-century Swedish physician. J Ethnobiol Ethnomed. (2025) 21:68. doi: 10.1186/s13002-025-00813-0 41121320 PMC12541932

[99] Sha AL . Effects of the *Fomes officinalis* flavonoids on anti-senile action in the aging model mice. Zhongguo Ying Yong Sheng Li Xue Za Zhi. (2016) 32:121–3. doi: 10.13459/j.cnki.cjap.2016.02.007 29931861

[100] Franceschi C Bonafè M Valensin S Olivieri F De Luca M Ottaviani E . Inflamm-aging: An evolutionary perspective on immunosenescence. Ann N Y Acad Sci. (2000) 908:244–54. doi: 10.1111/j.1749-6632.2000.tb06651.x 10911963

[101] Franceschi C Garagnani P Parini P Giuliani C Santoro A . Inflammaging: a new immune-metabolic viewpoint for age-related diseases. Nat Rev Endocrinol. (2018) 14:576–90. doi: 10.1038/s41574-018-0059-4 30046148

[102] Fülöp T Larbi A Pawelec G Khalil A Cohen AA Hirokawa K . Immunology of aging: The birth of inflammaging. Clinic Rev Allerg Immunol. (2023) 64:109–22. doi: 10.1007/s12016-021-08899-6 34536213 PMC8449217

[103] Fülöp T Larbi A Witkowski JM . Human inflammaging. Gerontology. (2019) 65:495–504. doi: 10.1159/000497375 31055573

[104] Franceschi C Capri M Monti D Giunta S Olivieri F Sevini F . Inflammaging and anti-inflammaging: A systemic perspective on aging and longevity emerged from studies in humans. Mech Ageing Dev. (2007) 128:92–105. doi: 10.1016/j.mad.2006.11.016 17116321

[105] Minciullo PL Catalano A Mandraffino G Casciaro M Crucitti A Maltese G . Inflammaging and anti-inflammaging: The role of cytokines in extreme longevity. Arch Immunol Ther Exp (Warsz). (2016) 64:111–26. doi: 10.1007/s00005-015-0377-3 26658771

[106] Plaza-Florido A Carrera-Bastos P Pérez-Prieto I Fiuza-Luces C Radom-Aizik S del Pozo Cruz B . The long-lived immune system of centenarians. Nat Rev Immunol. (2026) 26:1–19. doi: 10.1038/s41577-026-01291-5 42026253 PMC13747060

[107] Olivieri F Prattichizzo F Grillari J Balistreri CR . Cellular senescence and inflammaging in age-related diseases. Mediators Inflammation. (2018) 2018:9076485. doi: 10.1155/2018/9076485 29849499 PMC5932453

[108] Ovadya Y Krizhanovsky V . Senescent cells: SASPected drivers of age-related pathologies. Biogerontology. (2014) 15:627–42. doi: 10.1007/s10522-014-9529-9 25217383

