# Supplementary material for: Adaptive resilience: agarikon mycelium modulates immune responses and provides oxidative stress buffering in human immune cells
Source: Front Immunol. 2026 Sep 9;17:1946950. doi: 10.3389/fimmu.2026.1946950 (PMC13597427; doi:10.3389/fimmu.2026.1946950)
Supplement: Supplementary file 1 [file DataSheet1.docx]

Supplementary Material

**Supplementary Figure 1.** Representative images of agarikon- and vehicle-treated human immune cells (PBMCs) used for fluorometric cell proliferation assays under basal and LPS-induced inflammatory conditions.

**Supplementary Figure 2.** IL-8 cytokine responses in agarikon-treated human PBMCs under basal conditions (n = 4). Significance was evaluated relative to respective vehicle controls (PBS or DMSO) using two-tailed t-tests with unequal variances (**p* < 0.05; ***p* < 0.01).

**Supplementary Figure 3.** **(A)** Toll-like receptor (TLR) transcripts affected by agarikon treatment under basal and inflammatory conditions (n = 4, DESeq2, all TLR responses *p*adj < 0.05 included). **(B)** KEGG pathway for the TLR signaling pathway with genes affected by agarikon treatment in human PBMCs under basal and **(C)** inflammatory conditions highlighted according to average fold change (n = 4, DESeq2, *p*adj < 0.05).
